# Supplementary material for: Impacts of climate change, population growth, and power sector decarbonization on urban building energy use
Source: Nat Commun. 2023 Oct 18;14:6434. doi: 10.1038/s41467-023-41458-5 (PMC10584859; doi:10.1038/s41467-023-41458-5)
Supplement: Supplementary file 1 — Supplementary Information [file 41467_2023_41458_MOESM1_ESM.pdf]

Supplementary Information

Impacts of climate change, population growth, and power sector decarbonization on urban building energy use

Chenghao Wang[1,2,3]*, Jiyun Song[4,5,6], Dachuan Shi[4], Janet L. Reyna[7], Henry Horsey[7], Sarah Feron[8,9], Yuyu Zhou[5,10], Zutao Ouyang[1], Ying Li[11,12] & Robert B. Jackson[1,13,14]

[1]*Department of Earth System Science, Stanford University, Stanford, CA, USA*

[2]*School of Meteorology, University of Oklahoma, Norman, OK, USA*

[3]*Department of Geography and Environmental Sustainability, University of Oklahoma, Norman, OK, USA*

[4]*Department of Mechanical Engineering, The University of Hong Kong, Pokfulam Road, Hong Kong SAR, China*

[5]*Department of Geography, The University of Hong Kong, Pokfulam Road, Hong Kong SAR, China*

[6]*State Key Laboratory of Water Resources Engineering and Management, Wuhan University, Wuhan, China*

[7]*National Renewable Energy Laboratory, Golden, CO, USA*

[8]*Universidad de Santiago de Chile, Santiago, Chile*

[9]*University of Groningen, Groningen, The Netherlands*

[10]*Institute for Climate and Carbon Neutrality, The University of Hong Kong, Pokfulam Road, Hong Kong SAR, China*

[11]*Engineering Research Center of Eco-environment in Three Gorges Reservoir Region, Yichang, China*

[12]*College of Hydraulic and Environmental Engineering, China Three Gorges University, Yichang, China*

[13]*Woods Institute for the Environment, Stanford University, Stanford, CA, USA*

[14]*Precourt Institute for Energy, Stanford University, Stanford, CA, USA*

*Corresponding author. E-mail: chenghao.wang@ou.edu

Supplementary Method 1. A multi-step gap-filling approach for hourly weather data during the historical period

Although the vast majority of weather stations selected in this study are Class I or Class II stations, as classified in the Typical Meteorological Year (TMY3) dataset[1], some stations still have missing data during the historical period (1998–2019). To fill these data gaps, we developed a multi-step gap-filling approach. This approach primarily relies on statistical relationships between the station with missing data (hereafter the "target station"), the nearby station, and the nearby 4-km grid of the Modern-Era Retrospective Analysis for Research and Applications version 2 (MERRA-2) dataset[2]. The high-resolution MERRA-2 dataset used here was developed by the National Renewable Energy Laboratory and was included in the National Solar Radiation Database (NSRDB)[3].

We first identified the nearest station and the nearest MERRA-2 grid to each target weather station based on geographical distance. For air temperature, dew point temperature, surface pressure, and wind speed, we constructed locally adaptive regression models using the available data at the target station, the available data at the nearest station (within 220 km for temperatures and pressure as governed by similar mesoscale weather systems, but 50 km for wind speed to reflect local-scale effects), and the data at the nearest reanalysis data grid[4–7]. Note that information from the nearby station was introduced because a previous study[8] found that meteorological fields from the MERRA-2 dataset can be biased or slightly lagged when compared with station-based observations, mainly due to the differences in spatial resolution. Nevertheless, information from gridded datasets such as MERRA-2 is still valuable for regression-based gap filling[4].

To fill each missing data point, a multiple regression model was trained using data with varying moving window lengths, which depend on the extent of the data gap[9]. More specifically, for most stations, a 30-day (~a month) moving window centered at the missing data point was used[10]. To ensure statistical significance (p-value < 0.05) for regression models and that no more than 90% of data were missing at both stations, we further extended the moving window length to 90 days (~a season), a year, or three years when necessary. In cases where there were too many missing data points at both the target station and the nearby station, or when the multiple linear regression model was statistically insignificant, or when there was no nearby station within the specified search distance, we trained a simple linear regression model using the existing data

at the target station and data from the nearest MERRA-2 grid. For simple linear regression models, similar procedures (i.e., moving windows with different lengths) were followed as in multiple linear regression models to fill the data gap. In exceptionally rare instances, where there was an extensive amount of missing data and/or statistically insignificant regression models within the moving window, we directly replaced the missing data point with data from the nearest MERRA-2 grid. Note that hourly relative humidity was derived from the gap-filled air temperature and dew point temperature.

We also carried out quality control to identify and remove possible anomalously high or low values produced during the gap filling process using MERRA-2 gridded data, and correct variables to satisfy psychrometric relationships. Different from other variables, missing observations of wind direction were directly replaced with MERRA-2 data due to the poor performance of regression models. In addition, hourly precipitation data were often missing at many stations. Considering the relatively minor impact of precipitation data on building energy modeling (e.g., the green roof model in EnergyPlus is not used) and the uncertainties in statistical downscaling of future precipitation[11], we excluded liquid precipitation depth from historical and future weather data. Instead, the impact of precipitation events on temperature, humidity, cloud, radiation, etc., is reflected through other hourly weather variables.

Supplementary Method 2. Overview of the four SSPX-Y scenarios used in this study

The Intergovernmental Panel on Climate Change (IPCC)'s Sixth Assessment Report[12] used a core set of five illustrative scenarios based on the Shared Socio-economic Pathways (i.e., SSP1-1.9, SSP1-2.6, SSP2-4.5, SSP3-7.0, and SSP5-8.5). However, very few climate models produced 3-hourly outputs under the SSP1-1.9 scenario. This is mainly because this low emissions scenario is not among the four Tier 1 scenarios prioritized by the CMIP6's Scenario Model Intercomparison Project (ScenarioMIP)[13]. Therefore, we only consider SSP1-2.6, SSP2-4.5, SSP3-7.0, and SSP5-8.5 in this study, which already span a wide range of uncertainties in future socio-economic assumptions (such as land use, energy use, and population), emissions, climate change mitigations, and air pollution controls. These four scenarios also produce different future warming levels[14], as illustrated in Supplementary Table 2.

The name of each scenario is SSPX-Y, where X is the numbering of the SSP family that was used to develop the emissions pathway, and Y is the approximate radiative forcing reached by 2100 under this scenario. From the perspective of socio-economic development, the selected four SSP scenarios represent different combinations of future challenges related to climate change mitigation and adaptation[15]. SSP1 scenario, "Sustainability—Taking the Green Road", represents low challenges to mitigation and adaptation, under which the world shifts gradually (but pervasively) toward a more sustainable path. SSP2 scenario, "Middle of the road", represents medium challenges to mitigation and adaptation, under which the global social, economic, and technological patterns are not markedly different from historical ones. SSP3 scenario, "Regional rivalry—A rocky road", represents high challenges to mitigation and adaptation, under which countries are pushed to increasingly focus on domestic or regional issues. SSP5 scenario, "Fossil-fueled development—Taking the highway", represents low challenges to mitigation but high challenges to adaptation, under which the world focuses on producing rapid technological progress and human capital development toward sustainability with competitive markets, innovation, and participatory societies. A more detailed narrative and further information on each SSP can be found in refs.[15,16].

It is noteworthy that although some SSPX-Y scenarios in CMIP6 are continuations of previous Representative Concentration Pathways (RCP) scenarios[17] (RCP2.6, RCP4.5, RCP6.0, and RCP8.5), these SSPX-Y scenarios are different from the combinations of SSP socio-economic futures and RCP emissions and concentration futures under the SSPX-RCPY

framework commonly used in the literature[18,19]. On the one hand, compared with models in CMIP5, the latest generation of global climate models in CMIP6 has an improved representation of various physical processes with more realistic treatment of interactions within the climate system, and more models now have a numerical representation of biogeochemical cycles (some Earth system models are used in this study, see Supplementary Table 4). On the other hand, the outcomes of SSPX-Y scenarios in CMIP6 are also different from RCP scenarios in CMIP5. Supplementary Table 3 presents a comparison of four SSPX-Y scenarios used in this study and their closest RCP scenarios from ref.[12].

Supplementary Method 3. Special treatment for climate model outputs

While we tried to make sure that the selected climate models have 3-hourly outputs for essential variables (temperature, humidity, and radiation; some were added to the CMIP6 data repository in 2021 and 2022 by the modeling groups per our request), not all models output 3-hourly pressure and/or wind data (as of May 2022). For these models, we retrieved 6-hourly data as a substitute (Supplementary Table 4). More specifically:

(1) For CanESM5, 3-hourly surface air pressure data are not available under SSP2-4.5, SSP3-7.0, and SSP5-8.5 scenarios. We retrieved 6-hourly surface air pressure data as a substitute.

(2) For IITM-ESM, 3-hourly surface air pressure data are not available for the historical period and under all four SSPX-Y scenarios. We retrieved sea-level air pressure data with the highest temporal resolution under each scenario. For hindcast and projections under SSP1-2.6, 6-hourly sea-level air pressure was used, while for other SSPX-Y scenarios, daily sea-level air pressure was used. The sea-level air pressure data were then converted to surface pressure at stations.

(3) For FGOALS-g3, 3-hourly eastward near-surface wind and northward near-surface wind data are not available for the historical period and under all four SSPX-Y scenarios. As a substitute, we retrieved 6-hourly near-surface wind speed and 6-hourly eastward and northward wind speeds at the lowest (atmosphere) level in the model. The wind direction at 6-hourly resolution was then derived from the lowest model-level eastward and northward wind speeds as in the model. Note that the surface wind speed output from the model was diagnosed based on these two lowest model-level wind components and the Monin–Obukhov similarity theory.

We also retrieved daily maximum and minimum near-surface air temperature to better capture daily extreme temperatures which might be missing in the 3-hourly air temperature outputs (depending on the time zone). Daily maximum (minimum) temperature was inserted between the two highest (lowest) 3-hourly temperature data points to form a smooth diurnal cycle, although the exact hours with daily maximum and minimum temperatures might shift by one hour based on the knowledge gained from historical observations during 1998–2019 (the most likely hours with maximum and minimum temperatures). In practice, we found that the difference in extreme temperatures over a diurnal course between the daily dataset and the 3-hourly dataset is often very minor in most models.

In addition, according to our communication with the GISS-E2-1-G modeling team, the 3-hourly surface diffuse downwelling shortwave radiation diagnostic available via the CMIP6 data repository (variant ID: r1i1p1f2) was not properly output. A post hoc correction was done to fix this with the 3-hourly cosine of the solar zenith angle data from GISS-E2-1-G.

Supplementary Method 4. Prototype building simulations

Physics-based building energy modeling uses the residential and commercial prototype buildings[20,21] developed by the Pacific Northwest National Laboratory. For residential buildings, the official prototype building set has 32 building models for each of the 18 climate zones. These models cover two major types (single-family detached house and multi-family apartment building), four foundation types (vented crawlspace, slab-on-grade, heated basement, and unheated basement), and four heating system types (electric furnace, heat pump, gas-fired furnace, and oil-fired furnace). These prototype building models have been continuously updated to reflect changes in energy codes (International Energy Conservation Code; IECC) widely used in the country[20,22,23]. A detailed description of these residential building models, including building geometry, building envelope, internal gains, lighting, infiltration, Heating, Ventilation, and Air Conditioning (HVAC) systems, and domestic hot water system can be found in refs.[20,22]. Given that propane is among the major space heating energy types in the U.S. residential sector but is not covered by official prototype building models, we generated a set of 8 residential prototype buildings that use propane for space heating (four foundation types for each major residential type). Then for all station–city pairs included in this study, we have 12,120 residential prototype building models (40 archetypes × 303 station–city pairs).

For commercial buildings, the official prototype building set has 16 building models for each of the 19 climate zones: small office, medium office, large office, stand-alone retail, strip mall, primary school, secondary school, outpatient healthcare, hospital, small hotel, large hotel, warehouse (non-refrigerated), quick service restaurant, full service restaurant, mid-rise apartment, and high-rise apartment. Similar to residential prototype building models, commercial building models have also been updated frequently to incorporate changes in energy codes[21,24,25]. These commercial building models were originally derived from the commercial reference building models[26]. A detailed description of these models, such as building schedules, building forms, building envelope, occupancy, HVAC equipment, service water heating equipment, and lighting can be found in refs.[27,28]. It is noteworthy that mid-rise apartment and high-rise apartment, which are essentially multi-family buildings, were included as part of the commercial prototype building models because they are regulated by the IECC's commercial provisions[25]. To be consistent with the End-Use Load Profiles (EULP) (see Supplementary Method 5), the commercial building energy simulation in this study does not consider these two apartment

types. We expanded the 14 commercial building models into 28 types to take into account different heating types, i.e., each original prototype building can use natural gas or electricity for space heating. Therefore, for all station–city pairs we have 8,484 commercial prototype building models (28 archetypes × 303 station–city pairs). Note that for consistency, prototype building models developed based on the 2018 International Energy Conservation Code (IECC) were used for both commercial and residential sectors.

Supplementary Method 5. Further details of the End-Use Load Profiles (EULP) database, ResStock, and ComStock models

The EULP database uses high-granularity ResStock for the U.S. residential building stock. As a bottom-up, physics-based simulation model, ResStock uses a very large number of representative building models to represent the building stock of a region[29]. The characteristics of these representative building models are sampled from a network of conditional probability distributions of more than 100 building components (e.g., location, vintage, geometry, envelope, equipment, and occupant behavior) using a modified Latin hypercube sampling approach. The weight assigned to each representative building is also determined. These conditional probability distributions are constructed based on a synthesis of various data sources. For the EULP database, major data sources used to develop representative residential building models in ResStock[8] include the U.S. Census Bureau 2012–2016 American Community Survey (ACS) 5-year data[30], U.S. Census Bureau 2013–2017 ACS 5-year Public Use Microdata Sample[31], U.S. Energy Information Administration (EIA) 2009 Residential Energy Consumption Survey (RECS) data[32], 2015 RECS data[33], U.S. Census Bureau 2017 American Housing Survey (AHS) data[34], Northwest Energy Efficiency Alliance (NEEA) Residential Building Stock Assessment (RBSA) data (2011–2012)[35], NEEA RBSA II data (2016–2017)[36], 2009 International Energy Conservation Code (IECC)[37], ANSI/RESNET/ICC 301-2019 Standard[38], Homeland Infrastructure Foundation-Level Data (HIFLD) national parcel database (not available to the public), and the 2014 Building America House Simulation Protocols[39].

For the U.S. commercial building stock, the EULP database uses ComStock[40,41], another bottom-up, physics-based simulation model similar to ResStock. Major data sources used to construct a network of conditional probability distributions of building characteristics for ComStock[8] include U.S. Census Bureau 2013–2017 ACS 5-year Public Use Microdata Sample[31], U.S. EIA 2012 Commercial Buildings Energy Consumption Survey (CBECS) data[42], Homeland Security Infrastructure Program (HSIP) Gold 2012 database, 2017 CoStar Real Estate Data[43], 2015 U.S. Lighting Market Characterization[44], ASHRAE Service Life and Maintenance Cost Database[45], NEEA Commercial Building Stock Assessment (CBSA) 4 (2019)[46], ANSI/ASHRAE Standard 62.1 Ventilation for Acceptable Indoor Air Quality, ANSI/ASHRAE/IES Standard 90.1 Energy Standard for Buildings Except Low-Rise Residential Buildings, Building Codes Assistance Project[47], the Database for Energy Efficient Resources (DEER)[48], commercial

prototype building models[27,28], and different survey and submeter data. Note that both ResStock and ComStock models and their outputs have been used to examine the U.S. building stocks and their efficiency improvement[49–53].

It is noteworthy that a comprehensive validation or evaluation of the EULP outputs for all urban areas selected in our study is not possible, mainly limited by the lack of residential and commercial building energy use data with sufficient spatial (i.e., smaller than a county) and temporal (i.e., hourly) resolutions. Nevertheless, our confidence in using the EULP as the "ground truth data" for the proposed bottom-up approach mainly comes from the extensive calibration and validation efforts that have been undertaken[8,29]. A large amount of empirical ground truth data (both public and private) have been acquired to facilitate region-by-region calibration and multi-dimensional, multi-variable validation of the EULP database. For residential buildings, 18 data sources were used for calibration and validation, including national and state-level survey data on annual and monthly scales, regional and city-scale end-use metering data on the hourly scale, regional load research data on the hourly scale, and eight regional or city-scale hourly advanced metering infrastructure (AMI) datasets. The calibration and validation of commercial buildings also used 18 data sources, including national and state-level survey data on annual and monthly scales, regional and city-scale benchmarking datasets on the annual scale, ten regional or city-scale hourly AMI datasets, and multiple anonymous datasets. In particular, the AMI data cover more than 2.3 million meters in residential buildings and more than 0.2 million meters in ~60,000 commercial buildings. Detailed results of calibration and validation can be found in ref.[8].

Across the entire contiguous U.S. (CONUS), the EULP explicitly simulates 900,000 building models, of which 550,000 residential building models are used to represent 133.2 million residential dwelling units, and 350,000 commercial building models are used to represent 58 billion ft^2 of commercial buildings (1.8 million buildings)[8]. Note that the size of building samples has been determined through previous convergence tests[8,29]. Considering the raw input data sources and model validation results, the EULP dataset can roughly represent the average conditions of the CONUS residential and commercial building stocks in the last decade (2010–2019).

In order to train and test the calibration models in this study (see section Statistical calibration models in Methods), two complete annual runs are necessary. For residential

buildings, ResStock has completed three annual runs as of February 2022: 2018 actual meteorological year (AMY) run, 2019 AMY run, and typical meteorological year (TMY3) run, whereas for commercial buildings, ComStock only has two annual runs: 2018 AMY and TMY3 runs. Since one of the purposes of our calibration models is to correct the shape of annual load profiles and the response to similar weather patterns, we prioritize the use of AMY runs (driven by realistic weather data) over TMY3 runs (driven by a collection of monthly weather data from different years). Therefore, we used the two most recent runs (2019 AMY residential run and 2018 AMY commercial run; training datasets) to train the calibration models, and 2018 AMY residential run (2018 AMY) and TMY3 commercial run (testing datasets) for testing. For residential buildings, 2019 is the training period, and 2018 is the testing period, while for commercial buildings, 2018 is the training period, and TMY3 is the testing period.

For each annual dataset from the EULP, ResStock produces outputs for five residential building types: single-family detached, single-family attached, multi-family with 2–4 units, multi-family with 5+ units, and mobile homes, which is slightly different from the types in official residential prototype building models (Supplementary Method 4). Given the similarity among different dwelling units, when aggregating our raw building-level data to city-scale results, we used raw single-family detached results for the two single-family types and mobile homes in the EULP, and raw multi-family results for the two multi-family types in the EULP. Considering that we used different shares of building types, foundation types, and primary heating types in the city-scale building stock as the weighting factors to aggregate data[20,54], this treatment does produce city-scale results that match reasonably well with the EULP data for each residential building type. In addition, four major energy sources were considered in the EULP: electricity, natural gas, fuel oil, and propane, which is consistent with our residential prototype building simulations (Supplementary Method 4). Hourly wood consumption is not considered in the current version of EULP.

In comparison, ComStock produces outputs for 14 major commercial building types: small office, medium office, large office, retail (equivalent to stand-along retail), strip mall, primary school, secondary school, outpatient (equivalent to outpatient healthcare), hospital, small hotel, large hotel, warehouse, quick service restaurant, and full service restaurant. These 14 types are consistent with the commercial prototype building models (see Supplementary Method 4). ComStock simulates five major energy sources: electricity, natural gas, fuel oil, propane, and

district cooling/heating. However, in this study, we only consider electricity and natural gas for commercial buildings. This is because (1) the official commercial prototype building models are not capable of simulating fuel oil, propane, and district cooling/heating, and (2) for the selected 277 urban areas, on average the share of electricity and natural gas in commercial building energy use is 97.5%.

We aggregated the raw Public Use Microdata Areas (PUMAs) level 15-min EULP data to city-scale hourly data by major building types and end-uses based on the PUMA-city crosswalk (see section Selection of U.S. urban areas in Methods, Supplementary Fig. 26, and Supplementary Data 1). The end-use categories are summarized in Supplementary Table 5. Data outside the selected urban areas or PUMAs were excluded. The selected 277 urban areas cover ~84 million residential dwelling units (~63% of the CONUS residential building stock) with ~348,000 residential building models, and ~42.7 billion ft^2 of commercial buildings (~74% of the CONUS commercial building stock) with ~245,000 models.

Supplementary Method 6. Further details of the Cambium dataset and the calculation of regional site-to-source conversion factors for electricity

The Cambium dataset[55] contains hourly emission, energy cost, and operational data for the U.S. electric power sector under different future scenarios. It synthesizes structured outputs from three models: (1) the Regional Energy Deployment System (ReEDS) model[56], which models the generation, transmission, and end-use technologies of the electric power sector; (2) the Distributed Generation Market Demand (dGen) model[57], which projects the deployment of rooftop solar photovoltaics (PV); and (3) the PLEXOS[58], an energy modeling and forecasting software platform used to produce hourly outputs. For electricity generation, the Cambium version 2021 reports biennial projections (2022–2050) of 19 technology groups[55] consolidated from the original groups in the ReEDS and FLEXOS: coal (including scrubbed and un-scrubbed, integrated gasification combined cycle, and biomass cofired technologies), coal with carbon capture and storage, natural gas combined cycle, natural gas combined cycle with carbon capture and storage, natural gas combustion turbine, oil-gas-steam, nuclear (both conventional and small modular reactors), biomass (including biopower and landfill gas), bioenergy with carbon capture and storage, concentrating solar power, utility-scale PV and distributed-utility-scale PV, behind-the-meter PV, onshore wind, offshore wind, hydropower, pumped hydro storage, geothermal (including hydrothermal, near-field enhanced geothermal, and deep enhanced geothermal systems), electric batteries, and Canadian imports. The Cambium dataset has been commonly used to evaluate the impact of different building designs and retrofits[51,59,60].

The scenarios used in the Cambium dataset follow the annual Standard Scenarios developed by the National Renewable Energy Laboratory. The 7th Standard Scenarios[61] used in the Cambium version 2021 covers a wide range of 50 future scenarios of the U.S. power sector. In this study, we use two scenarios, the Mid-case scenario with no new CO_2 policy (after June 2021) and the Mid-case scenario with a 95% reduction in national power sector CO_2 emissions by 2035 and a 100% reduction by 2050 relative to 2005, which herein are referred to as *business-as-usual* and *zero-carbon* scenarios for convenience. Despite the difference in the decarbonization-related policy and regulatory environment, both scenarios adopt the demand growth rate and natural gas prices projected in the U.S. EIA's Annual Energy Output 2021 Reference case[62], and follow the 2021 Annual Technological Baseline moderate projections. The temporal changes in the national generation mix under these scenarios are shown in

Supplementary Fig. 18. It is clear that the share of natural gas and coal in generation under the zero-carbon scenario declines much faster than that under the business-as-usual scenario, accompanied by a rapid increase in the share of renewable energy technologies.

We used the generation mix and the associated heat rates of different primary energy sources in 2050 to estimate site-to-source conversion factors at the generation and emission assessment (GEA) level (Supplementary Fig. 35). For the historical period (2010–2019), we used the 2018 generation mix data from the Cambium v2020 dataset[63]. Supplementary Table 9 summarizes the heat rates we adopted to estimate conversion factors during historical and future periods. For non-combustible renewables, we follow the fossil fuel equivalency approach as in the U.S. EIA's Monthly Energy Review[64]. A fossil fuel equivalency factor was derived based on fossil fuel-fired generation and primary energy use[65]. The factor was then adopted to convert renewable electricity to equivalent source energy. We further applied a grid gross loss of 5% based on eGRID data[66,67] to account for transmission and distribution losses. To derive a site-to-source conversion factor for urban areas that span multiple GEAs, PUMA-level population data were used as weighting factors. This was done for both historical and future conversion factors, and as a result, conversion factors under different SSPX-Y scenarios can be different even for the same urban area.

Supplementary Method 7. Site-to-source conversion factors for fossil fuels

National level conversion factors are used to convert site consumption of natural gas, fuel oil, and propane to source energy consumption. Following ENERGY STAR[68], the site-to-source conversion factors for natural gas, fuel oil, and propane are 1.05, 1.01, and 1.01, respectively.

Supplementary Method 8. A modified elasticity-based decomposition method

We use an elasticity-based decomposition method to evaluate the potential uncertainties in the estimated contributions from climate change, population, and generation mix. This method was originally developed by ref.[69], and is modified in this study to consider the contributions of four different site building energy types.

We start from Eq. (13) in Methods. For each site energy type *i*, E_i, we have

$$E_{\mathrm{SO},i} = E_i \times PR \times CF_i \tag{S1}$$

where E_{SO} is the total source energy consumption of buildings in an urban area, *PR* is the population ratio defined in Methods (unity for the historical period; identical across all four site energy types), and *CF* is the site-to-source conversion factor.

The change of the source energy term can be written as

$$dE_{\mathrm{SO},i} = PR \times CF_i \times dE_i + E_i \times CF_i \times dPR + E_i \times PR \times dCF_i + \varepsilon \tag{S2}$$

where ε is the sum of all higher-order terms.

Dividing both sides of Eq. (S2) by $E_{\mathrm{SO},i}$ leads to

$$\frac{dE_{\mathrm{SO},i}}{E_{\mathrm{SO},i}} = \frac{dE_i}{E_i} + \frac{dPR}{PR} + \frac{dCF_i}{CF_i} + \varepsilon' \tag{S3}$$

Eq. (S3) is essentially similar to the method used in ref.[70].

We then rearrange Eq. (S3) by inserting $dE_{\mathrm{SO},i} / E_{\mathrm{SO},i}$ on the right-hand side

$$dE_{\mathrm{SO},i} = \frac{dE_i / E_i}{dE_{\mathrm{SO},i} / E_{\mathrm{SO},i}} dE_{\mathrm{SO},i} + \frac{dPR / PR}{dE_{\mathrm{SO},i} / E_{\mathrm{SO},i}} dE_{\mathrm{SO},i} + \frac{dCF_i / CF_i}{dE_{\mathrm{SO},i} / E_{\mathrm{SO},i}} dE_{\mathrm{SO},i} + \varepsilon'' \tag{S4}$$

The equation above suggests that the change in source energy can be attributed using an elasticity ratio. Eq. (S4) can be rewritten in a similar form as Eq. (14), but with an intrinsic residual term

$$\Delta E_{\mathrm{SO}}^{\mathrm{ELAS}} = \Delta E_{E}^{\mathrm{ELAS}} + \Delta E_{PR}^{\mathrm{ELAS}} + \Delta E_{CF}^{\mathrm{ELAS}} + \varepsilon''' \tag{S5}$$

where

$$\Delta E_{E}^{\mathrm{ELAS}} = \frac{\left(E_{i,\mathrm{fut}} - E_{i,\mathrm{his}}\right) / A\left(E_{i,\mathrm{fut}}, E_{i,\mathrm{his}}\right)}{\left(E_{\mathrm{SO},i,\mathrm{fut}} - E_{\mathrm{SO},i,\mathrm{his}}\right) / A\left(E_{\mathrm{SO},i,\mathrm{fut}}, E_{\mathrm{SO},i,\mathrm{his}}\right)} \left(E_{\mathrm{SO},i,\mathrm{fut}} - E_{\mathrm{SO},i,\mathrm{his}}\right) \tag{S6}$$

$$\Delta E_{PR}^{\mathrm{ELAS}} = \frac{\left(PR_{i,\mathrm{fut}} - PR_{i,\mathrm{his}}\right) / A\left(PR_{i,\mathrm{fut}}, PR_{i,\mathrm{his}}\right)}{\left(E_{\mathrm{SO},i,\mathrm{fut}} - E_{\mathrm{SO},i,\mathrm{his}}\right) / A\left(E_{\mathrm{SO},i,\mathrm{fut}}, E_{\mathrm{SO},i,\mathrm{his}}\right)} \left(E_{\mathrm{SO},i,\mathrm{fut}} - E_{\mathrm{SO},i,\mathrm{his}}\right) \tag{S7}$$

$$\Delta E_{CF}^{\mathrm{ELAS}} = \frac{\left(CF_{i,\mathrm{fut}} - CF_{i,\mathrm{his}}\right) / A\left(CF_{i,\mathrm{fut}}, CF_{i,\mathrm{his}}\right)}{\left(E_{\mathrm{SO},i,\mathrm{fut}} - E_{\mathrm{SO},i,\mathrm{his}}\right) / A\left(E_{\mathrm{SO},i,\mathrm{fut}}, E_{\mathrm{SO},i,\mathrm{his}}\right)} \left(E_{\mathrm{SO},i,\mathrm{fut}} - E_{\mathrm{SO},i,\mathrm{his}}\right) \quad \text{(S8)}$$

In Eqs. (S5)–(S8), the superscript "ELAS" denotes the elasticity-based approach, while *A* means arithmetic average.

Supplementary Fig. 36 shows the difference between the estimated contributions using the logarithmic mean Divisia index (LMDI) method and the elasticity-based approach ($\Delta E^{\mathrm{ELAS}} - \Delta E^{\mathrm{LMDI}}$). It is clear that the discrepancies in the estimates using these two methods are marginal. Under all four SSPX-Y scenarios, the relative discrepancies, $(\Delta E^{\mathrm{ELAS}} - \Delta E^{\mathrm{LMDI}}) / \Delta E^{\mathrm{LMDI}}$, range from –1.02% to 0.57% and from –1.59% to 0.14% for business-as-usual and zero-carbon scenarios, respectively.

Supplementary Note 1. The use of “site” in the captions of supplementary figures

To be consistent with the main text, we simplify “site energy use” as “energy use” in the captions of all supplementary figures, unless otherwise specified. This includes energy use intensity, which is calculated using site energy use.

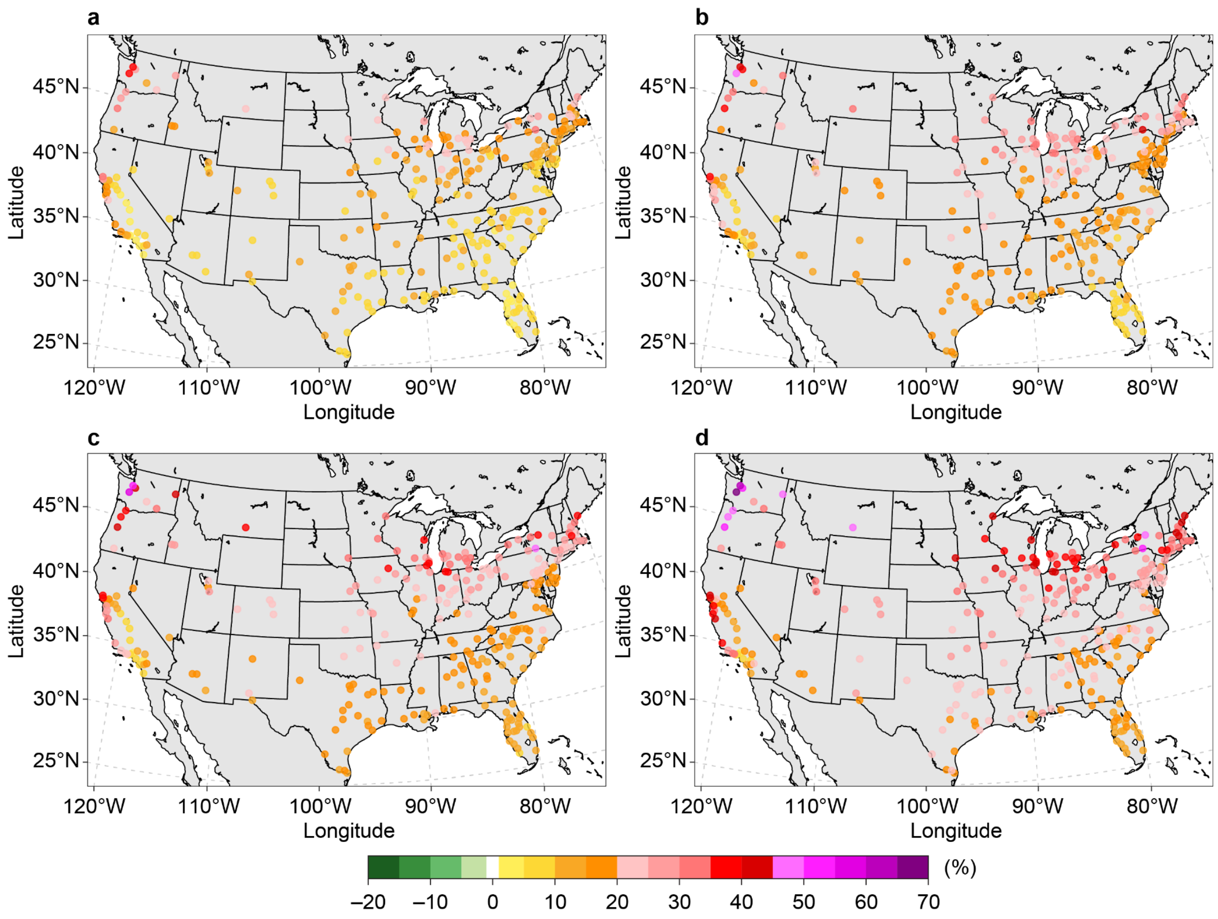

Supplementary Fig. 1. Change in annual electricity energy use intensity for cooling in the 2050s relative to the reference decade (the 2010s) under four illustrative SSPX-Y scenarios. **a,** SSP1-2.6 scenario. **b,** SSP2-4.5 scenario. **c**, SSP3-7.0 scenario. **d,** SSP5-8.5 scenario. Each point represents the relative change (%) based on the ensemble mean of the simulations driven by 10 CMIP6 models under each SSPX-Y scenario. Sources of base map: U.S. Census Bureau and Natural Earth.

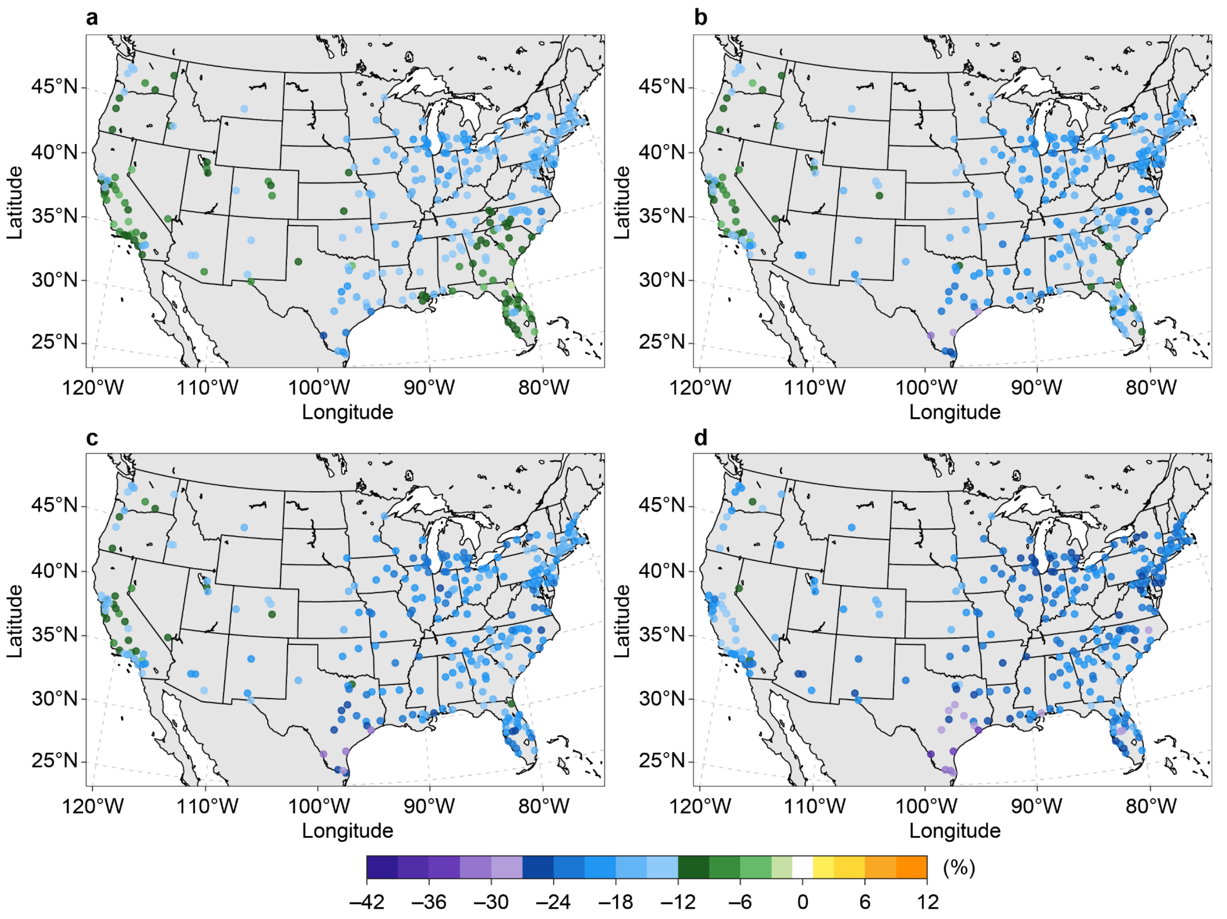

Supplementary Fig. 2. Change in annual electricity energy use intensity for space heating in the 2050s relative to the reference decade under four illustrative SSPX-Y scenarios. a, SSP1-2.6 scenario. **b,** SSP2-4.5 scenario. **c**, SSP3-7.0 scenario. **d,** SSP5-8.5 scenario. Each point represents the relative change (%) based on the ensemble mean of the simulations driven by 10 CMIP6 models under each SSPX-Y scenario. Sources of base map: U.S. Census Bureau and Natural Earth.

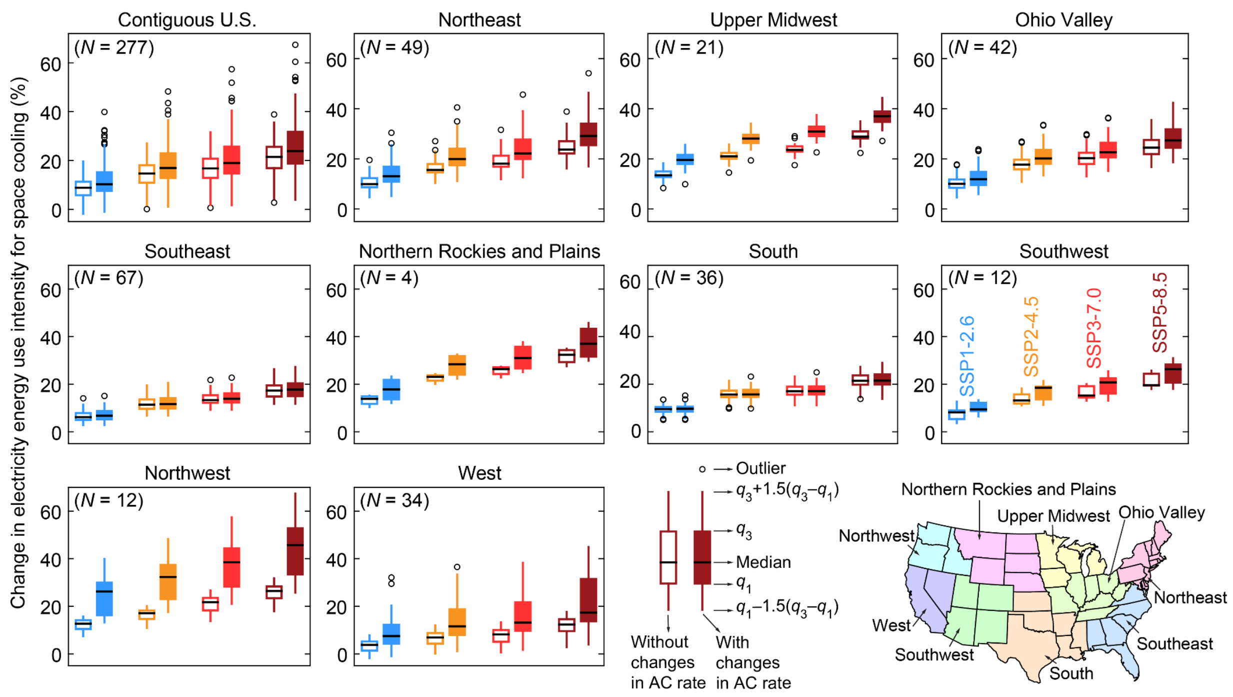

Supplementary Fig. 3. Influence of increasing city-scale air-conditioning saturation rate on the change in electricity energy use intensity for cooling in the 2050s relative to the reference decade in the CONUS and nine climate regions. Boxes in each subplot show the intercity variability of national or regional changes; unfilled and filled boxes represent results without and with changing air-conditioning (AC) saturation rate in the 2050s, respectively. In each box plot, the center line shows median, the box limits are upper quartile (q_3) and lower quartile (q_1), the whiskers are 1.5 × interquartile range, and the points are outliners. *N* in each subplot denotes the number of urban areas in each region. The inset map shows the division of nine climate regions according to the National Centers for Environmental Information[71]. Source of base map: U.S. Census Bureau.

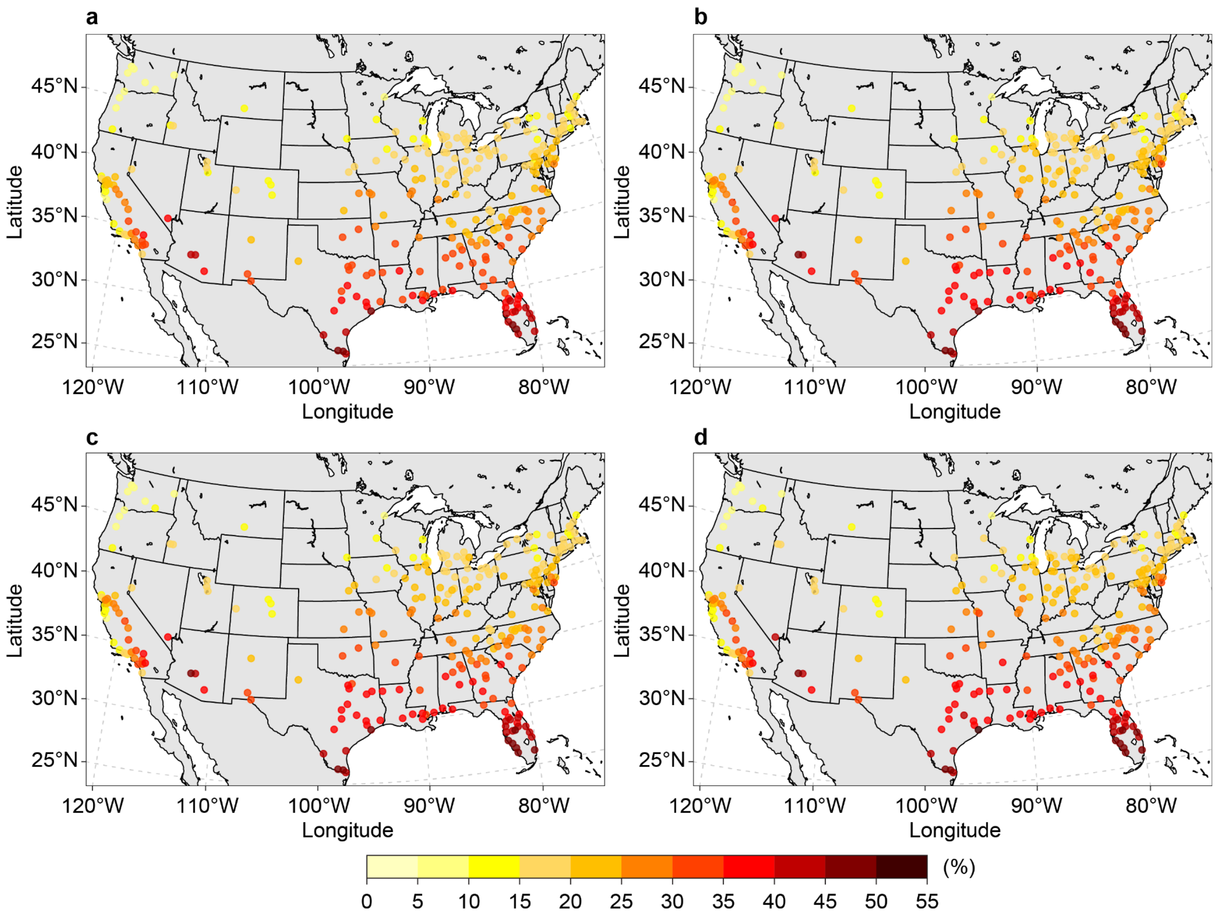

Supplementary Fig. 4. Share of annual electricity energy use intensity for cooling in the 2050s under four illustrative SSPX-Y scenarios. a, SSP1-2.6 scenario. **b,** SSP2-4.5 scenario. **c**, SSP3-7.0 scenario. **d,** SSP5-8.5 scenario. Each point represents the share (%) in total electricity energy use intensity based on the ensemble mean of the simulations driven by 10 CMIP6 models under each SSPX-Y scenario. Sources of base map: U.S. Census Bureau and Natural Earth.

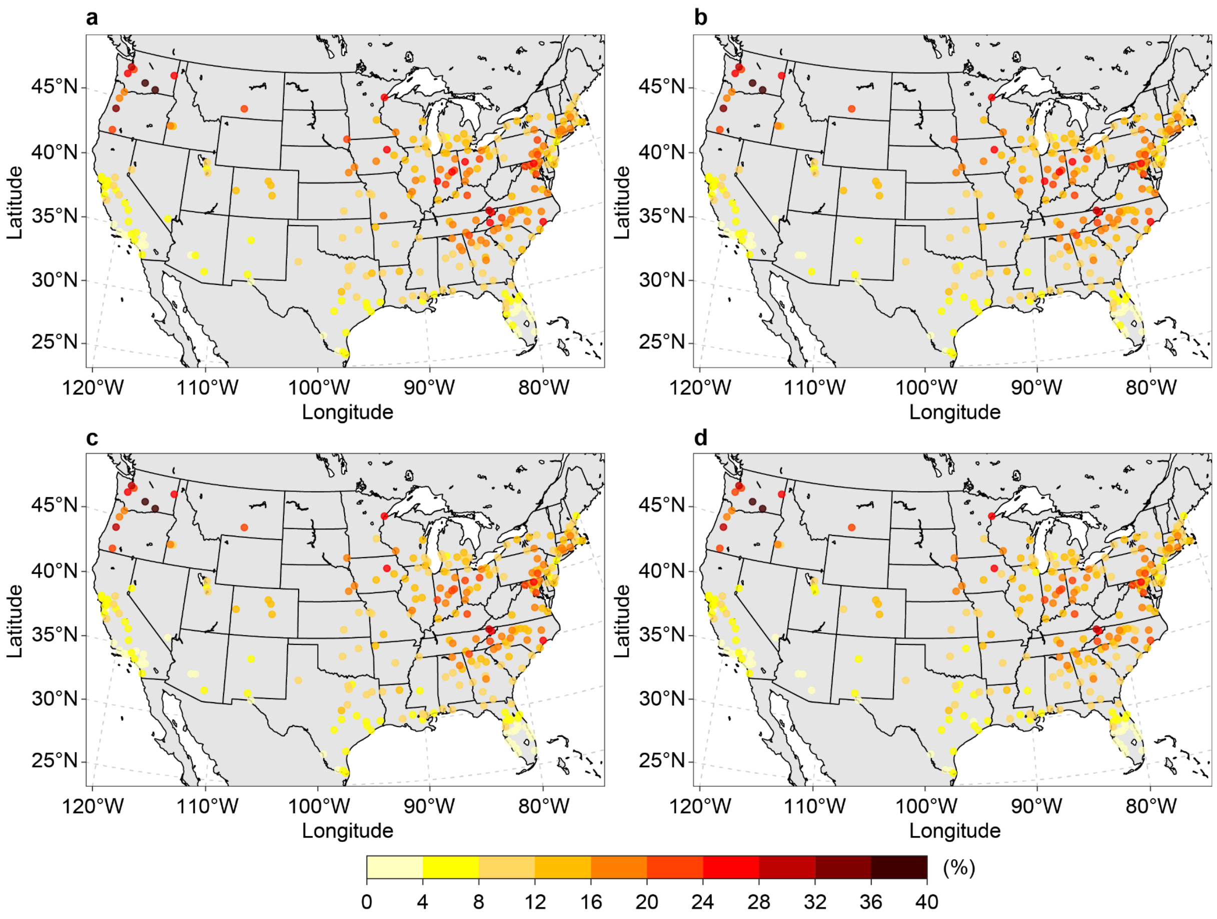

Supplementary Fig. 5. Share of annual electricity energy use intensity for space heating in the 2050s under four illustrative SSPX-Y scenarios. a, SSP1-2.6 scenario. **b,** SSP2-4.5 scenario. **c**, SSP3-7.0 scenario. **d,** SSP5-8.5 scenario. Each point represents the share (%) in total electricity energy use intensity based on the ensemble mean of the simulations driven by 10 CMIP6 models under each SSPX-Y scenario. Sources of base map: U.S. Census Bureau and Natural Earth.

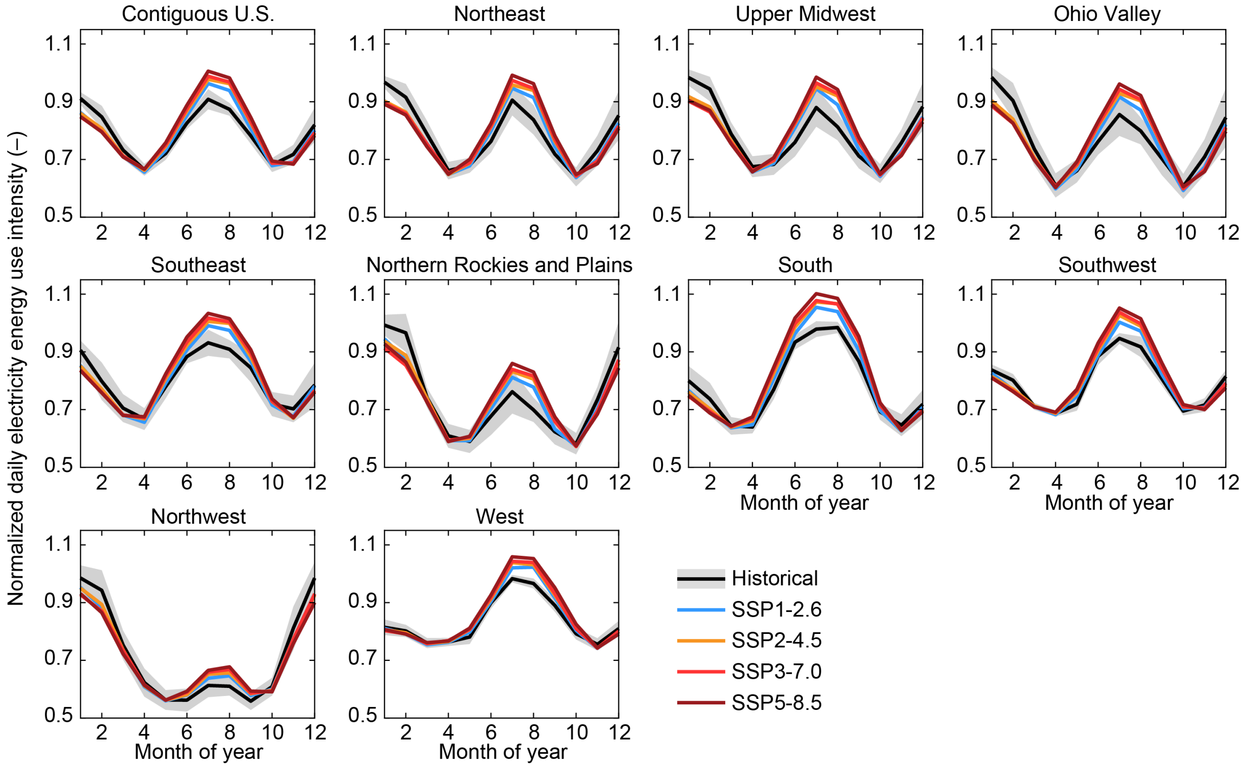

Supplementary Fig. 6. Monthly variation of historical (the 2010s) and future (the 2050s) city-scale daily electricity energy use intensity in the CONUS and nine climate regions. For comparison, the monthly profile of each urban area is normalized by the corresponding mean maximum monthly electricity energy use intensity. Error bands represent the interannual variability (± 1 standard deviation) during the historical period for each region.

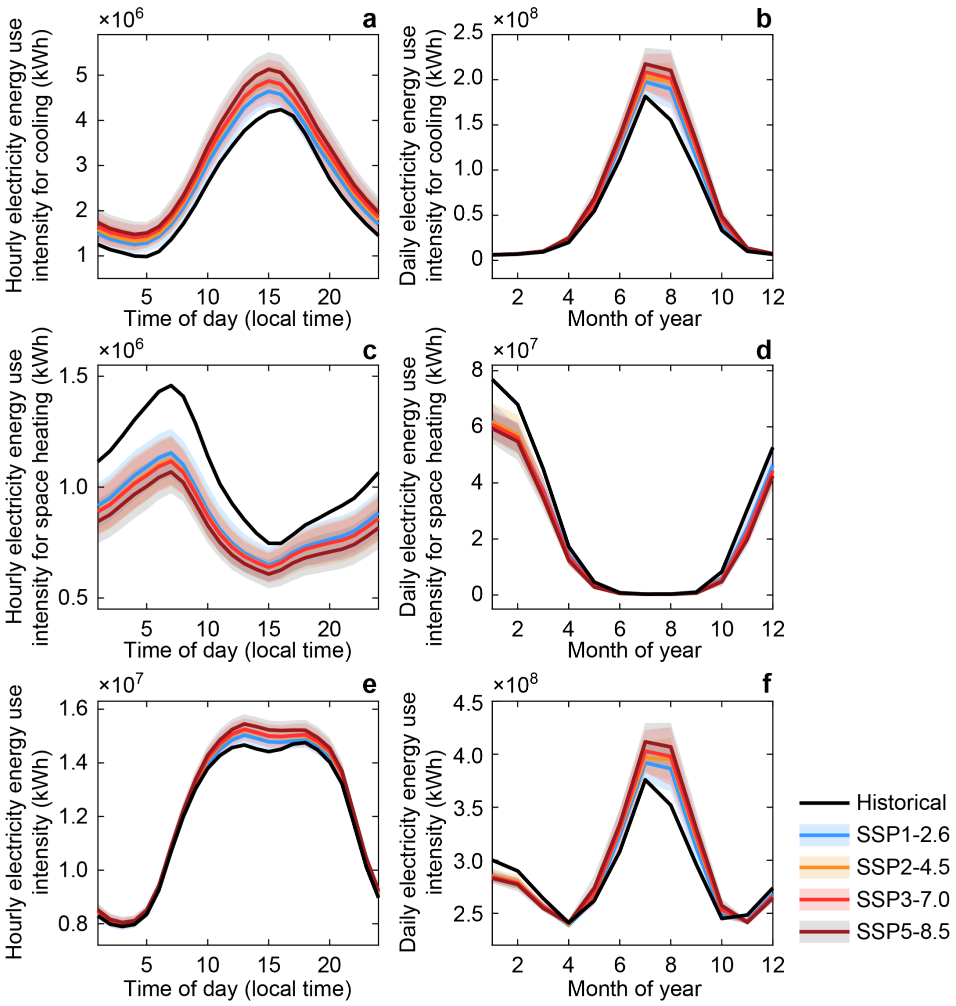

Supplementary Fig. 7. Diurnal and monthly variation of historical (the 2010s) and future (the 2050s) city-scale electricity energy use intensity in New York-Newark urban area. a, Diurnal variation of hourly electricity energy use intensity for cooling. **b,** Monthly variation of daily electricity energy use intensity for cooling. **c,** Diurnal variation of hourly electricity energy use intensity for space heating. **d,** Monthly variation of daily electricity energy use intensity for space heating. **e,** Diurnal variation of hourly electricity energy use intensity. **f,** Monthly variation of daily electricity energy use intensity. Data for each SSPX-Y scenario are presented as mean values ± 1 standard deviation of 10 ensemble members (driven by different climate models).

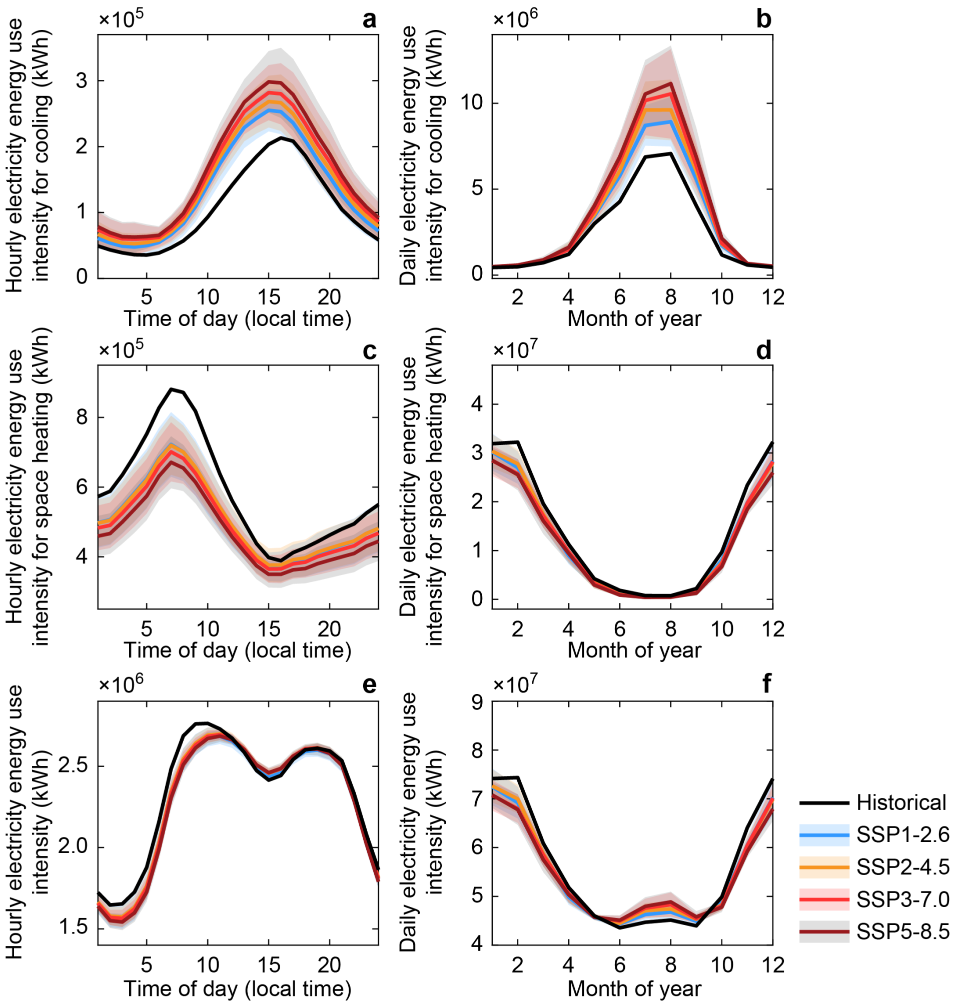

Supplementary Fig. 8. Diurnal and monthly variation of historical (the 2010s) and future (the 2050s) city-scale electricity energy use intensity in Seattle urban area. a, Diurnal variation of hourly electricity energy use intensity for cooling. **b,** Monthly variation of daily electricity energy use intensity for cooling. **c,** Diurnal variation of hourly electricity energy use intensity for space heating. **d,** Monthly variation of daily electricity energy use intensity for space heating. **e,** Diurnal variation of hourly electricity energy use intensity. **f,** Monthly variation of daily electricity energy use intensity. Data for each SSPX-Y scenario are presented as mean values ± 1 standard deviation of 10 ensemble members (driven by different climate models).

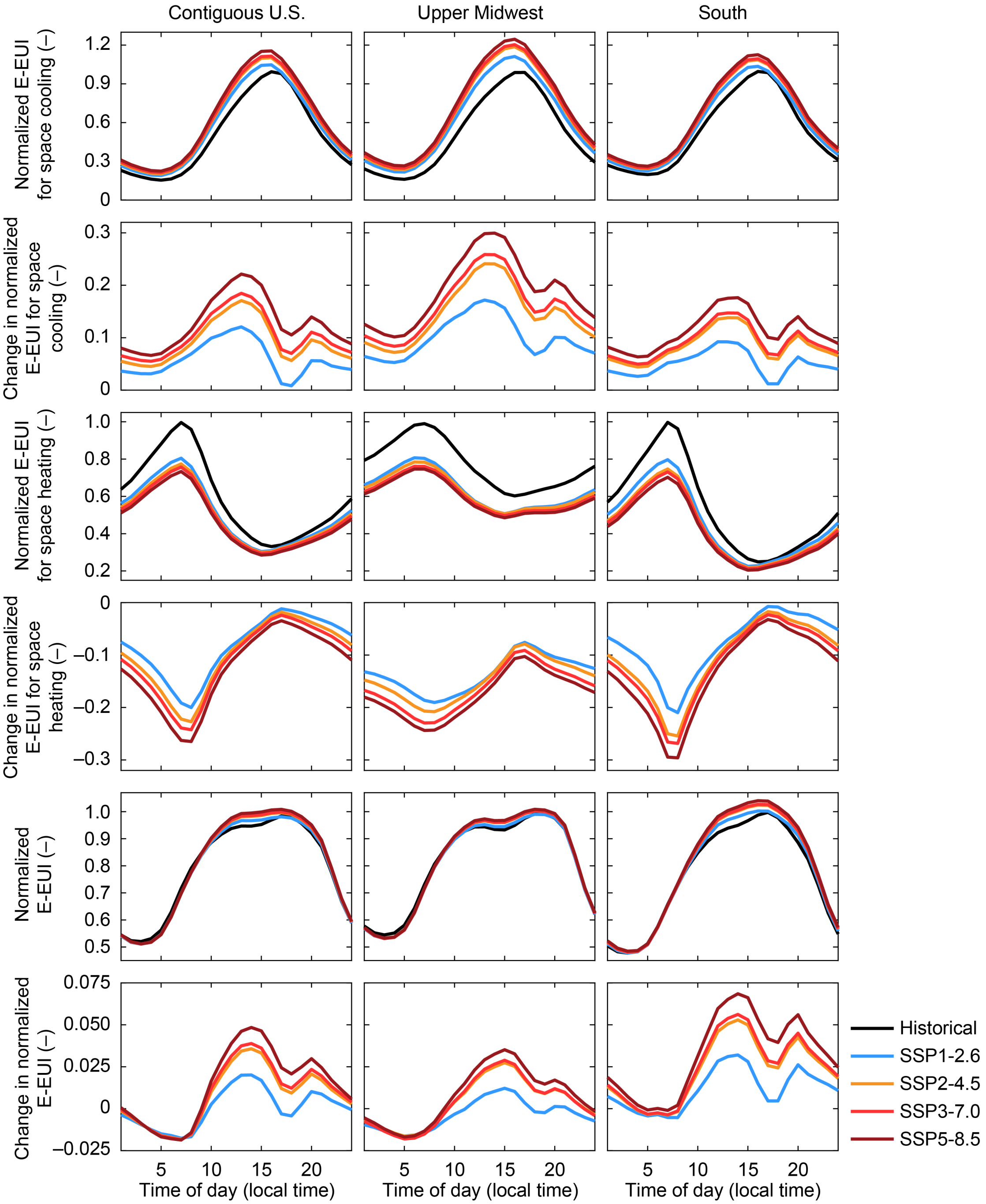

Supplementary Fig. 9. Diurnal variation of historical (the 2010s) and future (the 2050s) city-scale electricity energy use intensity and its changes in the CONUS (left), Upper Midwest (middle), and South (right). For comparison, the diurnal profile of each hourly variable (cooling, space heating, or total) in each urban area is normalized by the mean maximum hourly electricity energy use intensity to yield normalized electricity energy use intensity (E-EUI).

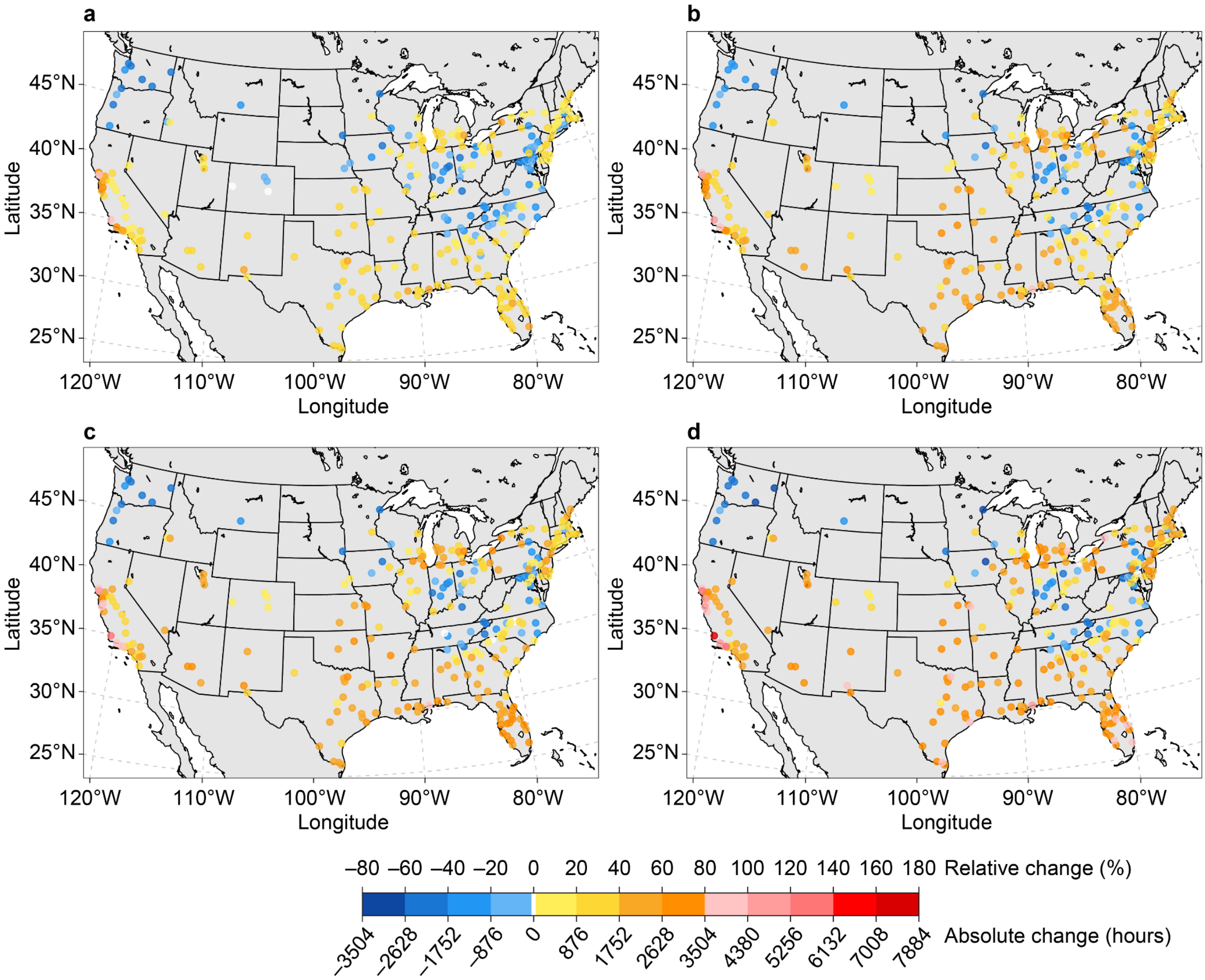

Supplementary Fig. 10. Change in the number of peak hours for electricity energy use intensity in the 2050s relative to the reference decade under four illustrative SSPX-Y scenarios. a, SSP1-2.6 scenario. **b,** SSP2-4.5 scenario. **c**, SSP3-7.0 scenario. **d,** SSP5-8.5 scenario. Each point represents the relative change (%) based on the ensemble mean of the simulations driven by 10 CMIP6 models under each SSPX-Y scenario. Here the number of peak hours is used to indicate peak demand frequency. Peak hours are defined as hours with electricity energy use intensity higher than the current (the reference decade) 95th percentile of electricity energy use intensity. Based on this definition, the number of peak hours in each urban area during the reference decade is 4380. Sources of base map: U.S. Census Bureau and Natural Earth.

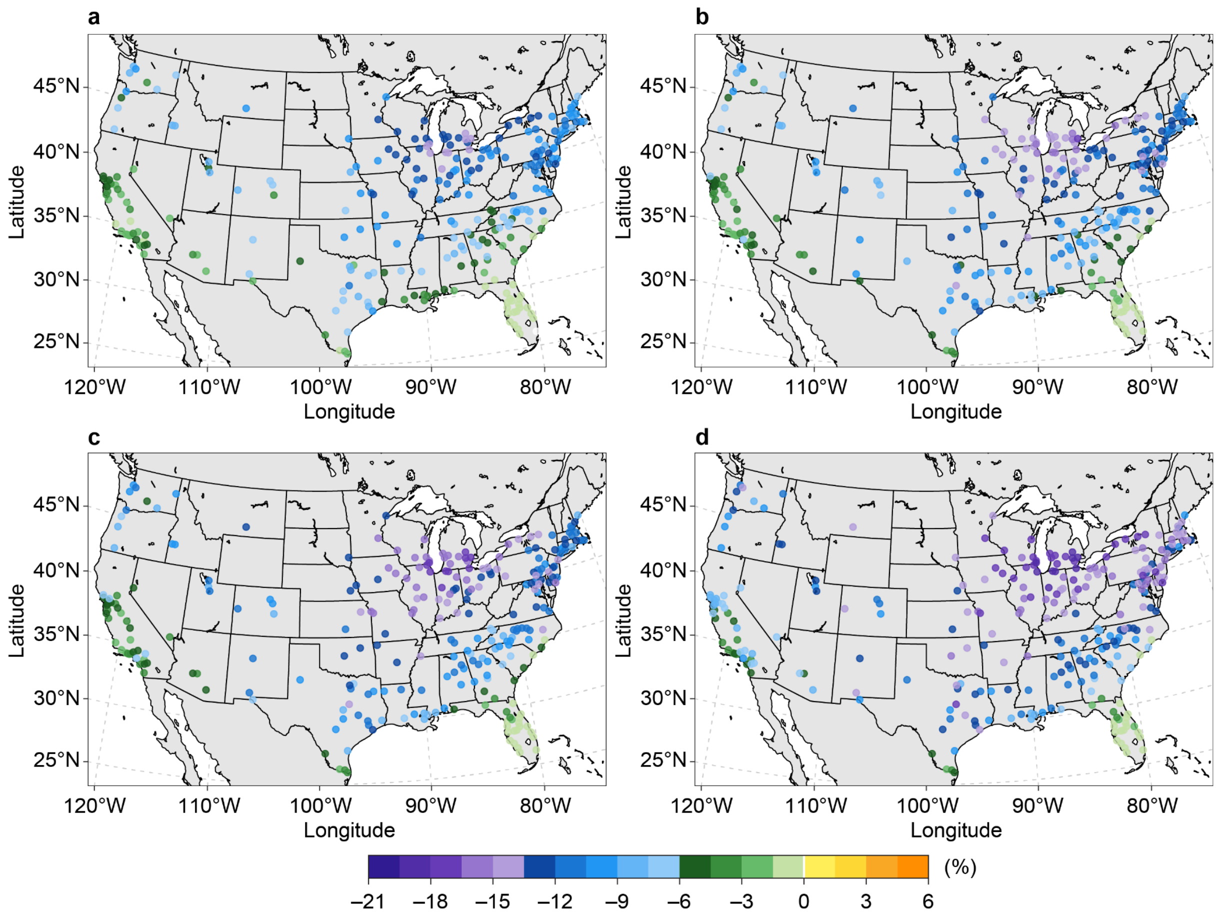

Supplementary Fig. 11. Change in annual natural gas energy use intensity in the 2050s relative to the reference decade under four illustrative SSPX-Y scenarios. a, SSP1-2.6 scenario. **b,** SSP2-4.5 scenario. **c**, SSP3-7.0 scenario. **d,** SSP5-8.5 scenario. Each point represents the relative change (%) based on the ensemble mean of the simulations driven by 10 CMIP6 models under each SSPX-Y scenario. Sources of base map: U.S. Census Bureau and Natural Earth.

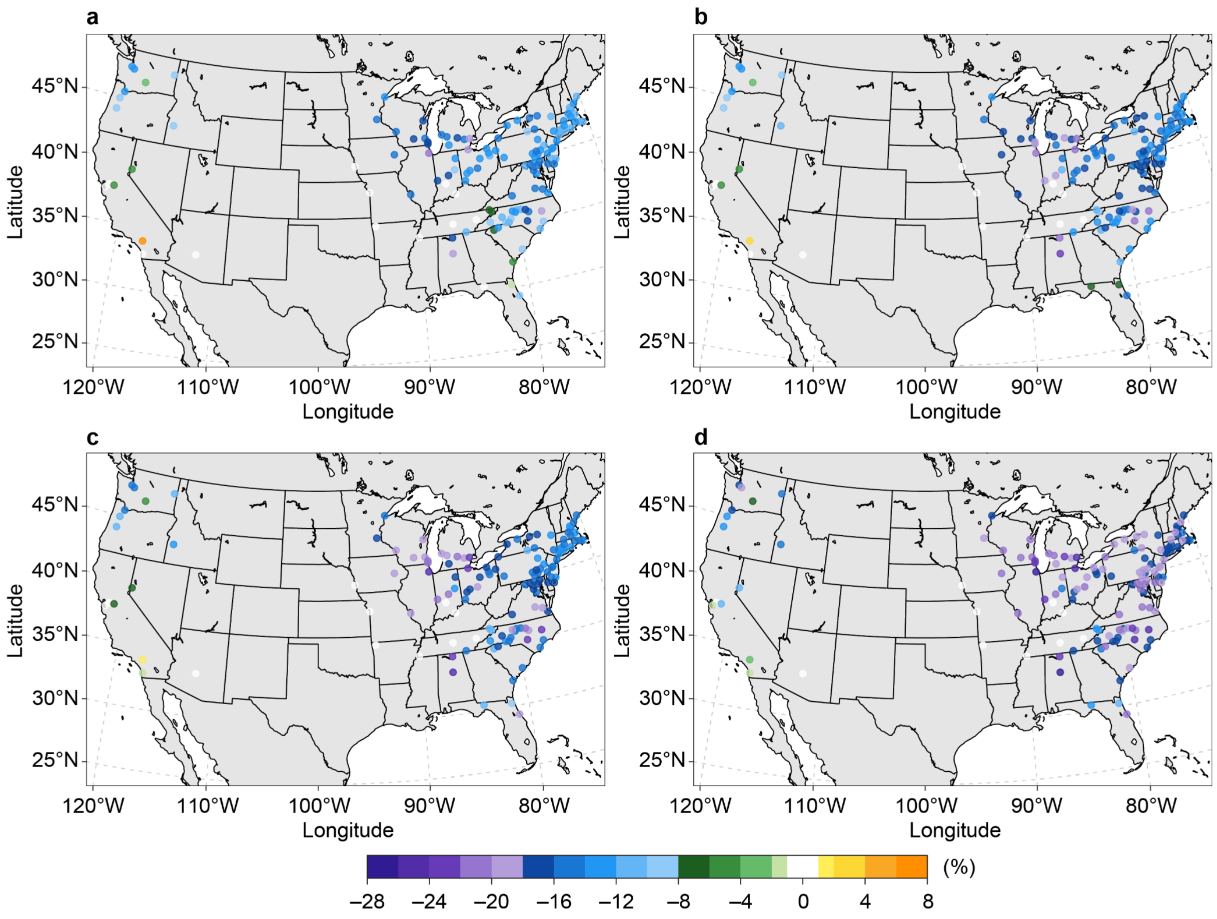

Supplementary Fig. 12. Change in annual fuel oil energy use intensity in the 2050s relative to the reference decade under four illustrative SSPX-Y scenarios. a, SSP1-2.6 scenario. **b,** SSP2-4.5 scenario. **c**, SSP3-7.0 scenario. **d,** SSP5-8.5 scenario. Each point represents the relative change (%) based on the ensemble mean of the simulations driven by 10 CMIP6 models under each SSPX-Y scenario. Sources of base map: U.S. Census Bureau and Natural Earth.

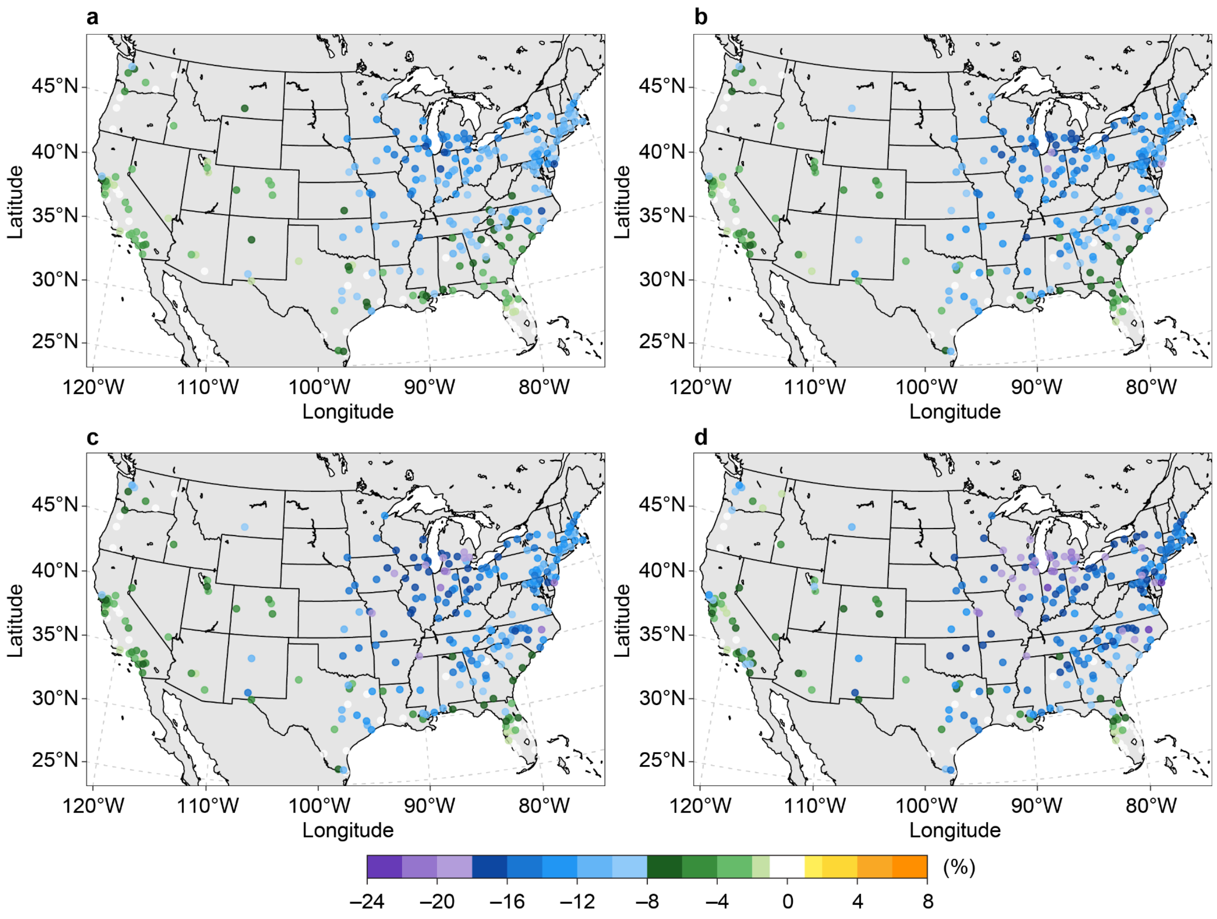

Supplementary Fig. 13. Change in annual propane energy use intensity in the 2050s relative to the reference decade under four illustrative SSPX-Y scenarios. a, SSP1-2.6 scenario. **b,** SSP2-4.5 scenario. **c**, SSP3-7.0 scenario. **d,** SSP5-8.5 scenario. Each point represents the relative change (%) based on the ensemble mean of the simulations driven by 10 CMIP6 models under each SSPX-Y scenario. Sources of base map: U.S. Census Bureau and Natural Earth.

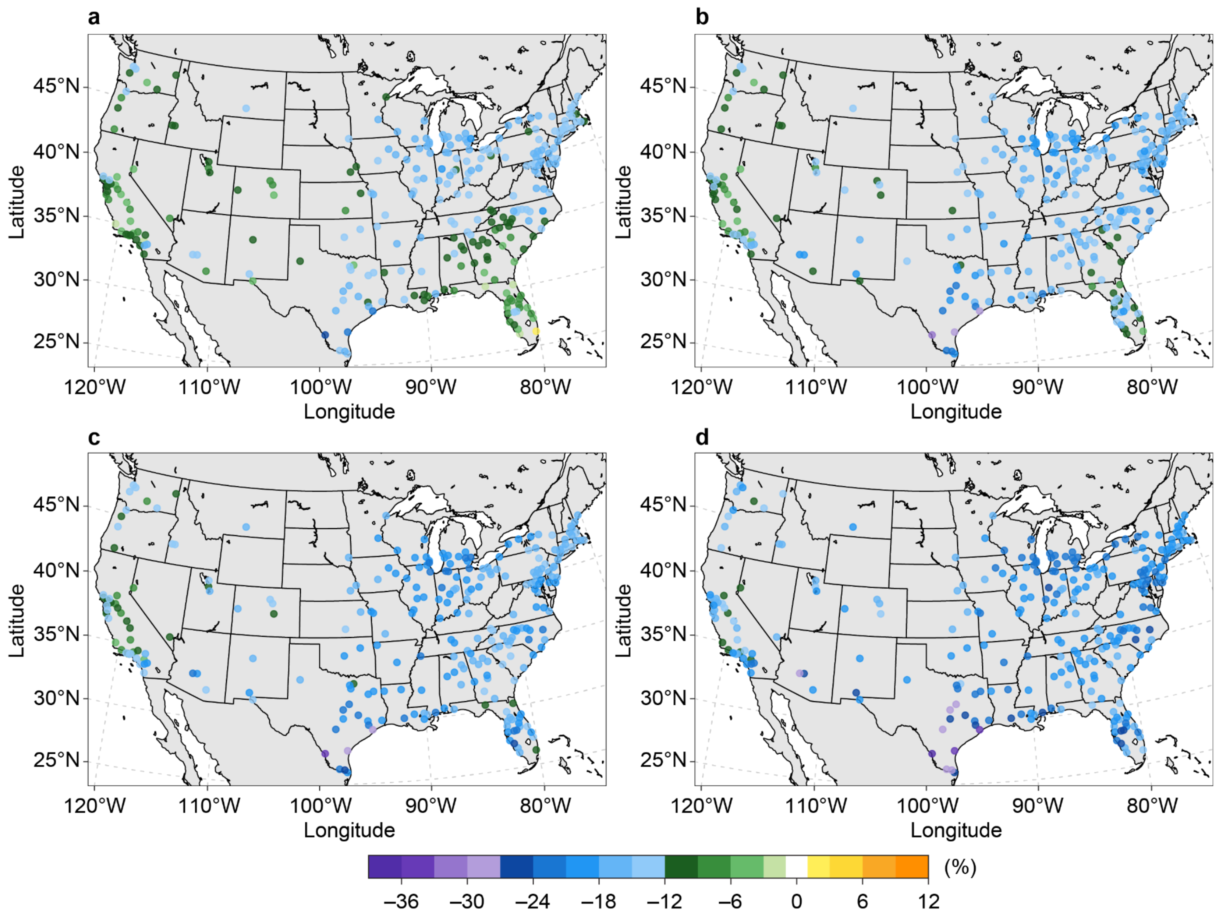

Supplementary Fig. 14. Change in annual fossil fuel energy use intensity for space heating in the 2050s relative to the reference decade under four illustrative SSPX-Y scenarios. a, SSP1-2.6 scenario. **b,** SSP2-4.5 scenario. **c**, SSP3-7.0 scenario. **d,** SSP5-8.5 scenario. Types of fossil fuels consumed by buildings include natural gas, fuel oil, and propane. Each point represents the relative change (%) based on the ensemble mean of the simulations driven by 10 CMIP6 models under each SSPX-Y scenario. Sources of base map: U.S. Census Bureau and Natural Earth.

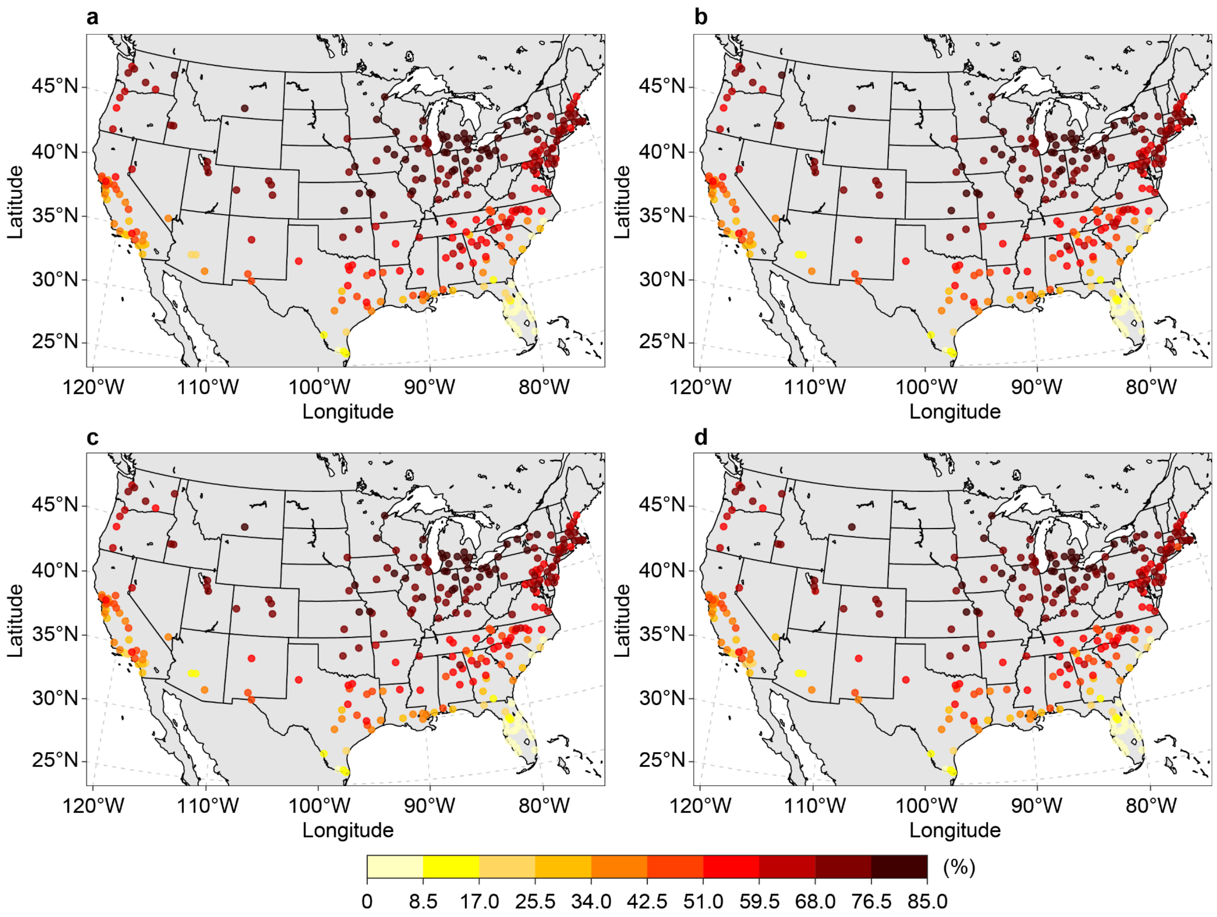

Supplementary Fig. 15. Share of annual natural gas energy use intensity for space heating in the 2050s under four illustrative SSPX-Y scenarios. a, SSP1-2.6 scenario. **b,** SSP2-4.5 scenario. **c**, SSP3-7.0 scenario. **d,** SSP5-8.5 scenario. Each point represents the share (%) in total natural gas energy use intensity based on the ensemble mean of the simulations driven by 10 CMIP6 models under each SSPX-Y scenario. Sources of base map: U.S. Census Bureau and Natural Earth.

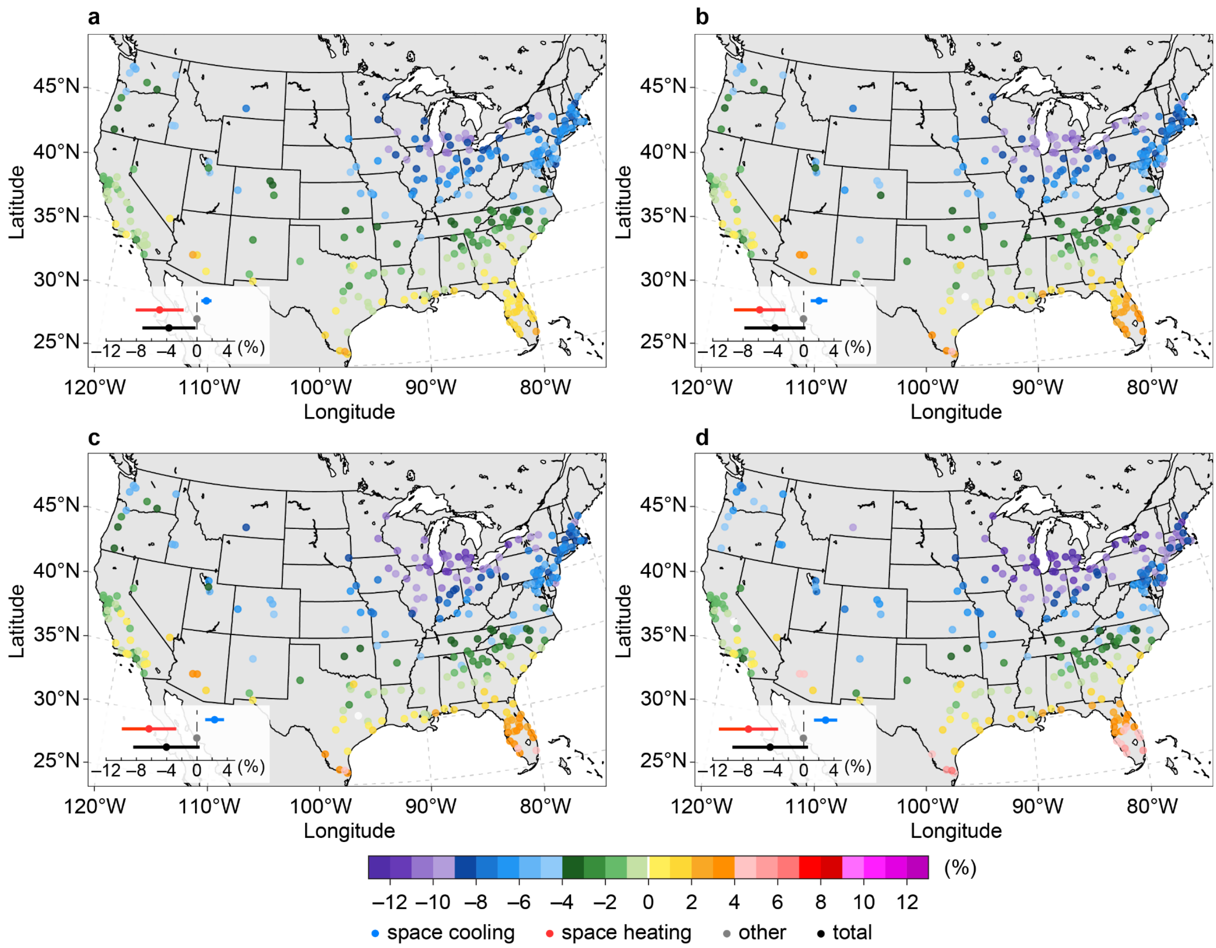

Supplementary Fig. 16. Change in annual total energy use intensity in the 2050s relative to the reference decade under four illustrative SSPX-Y scenarios. a, SSP1-2.6 scenario. **b,** SSP2-4.5 scenario. **c**, SSP3-7.0 scenario. **d,** SSP5-8.5 scenario. Each point represents the relative change (%) based on the ensemble mean of the simulations driven by 10 CMIP6 models under each SSPX-Y scenario. Inset shows the total relative change decomposed into cooling change, space heating change, and change in the energy consumption of other appliances, averaged over all CONUS urban areas, with error bars representing the variability (± 1 standard deviation) among urban areas ($N = 277$). Note that the length of error bars for the "other" category is virtually zero. Sources of base map: U.S. Census Bureau and Natural Earth.

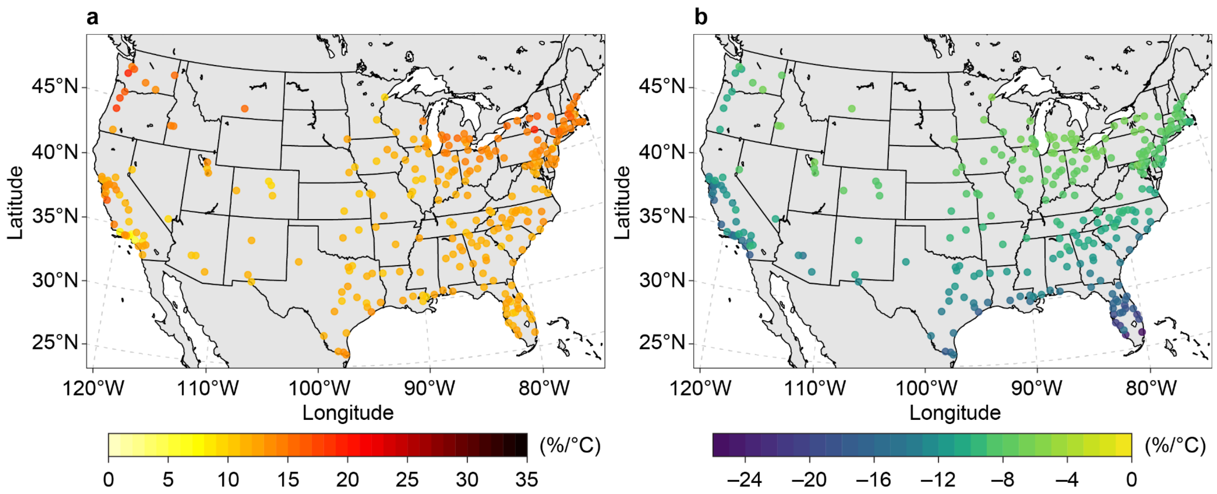

Supplementary Fig. 17. Response of annual electricity energy use intensity for cooling, with fixed air-conditioning saturation rate, and natural gas energy use intensity for space conditioning to a 1°C temperature change in the 2050s relative to the reference decade. a, Response of annual electricity energy use intensity for cooling with fixed air-conditioning saturation rate. **b,** Response of annual natural gas energy use intensity for space heating. Each point represents the response of energy use intensity to warming evaluated as the slope (%/°C) of the linear regression fit to all simulations under all SSPX-Y scenarios. Sources of base map: U.S. Census Bureau and Natural Earth.

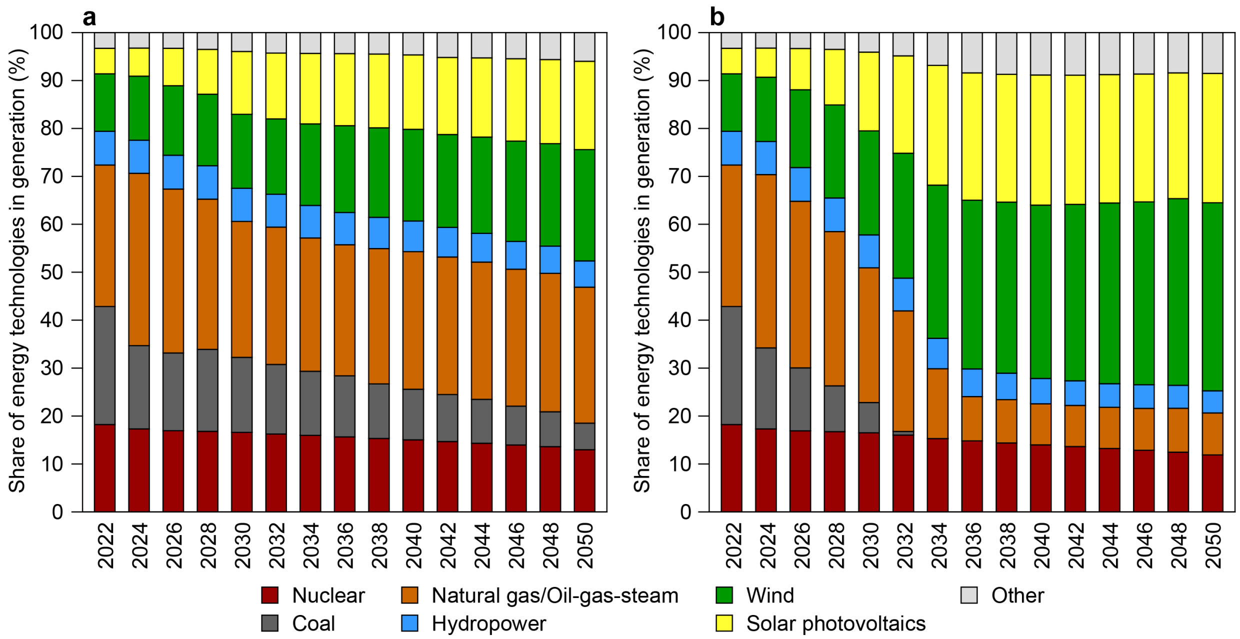

Supplementary Fig. 18. Change of electricity generation mix in the U.S. electric power sector over time under two scenarios. a, Business-as-usual scenario. **b,** Zero-carbon scenario. Note that under the zero-carbon scenario, emissions from fuel combustion are offset by negative emission technologies, such as carbon capture and sequestration and direct air capture, to achieve net zero.

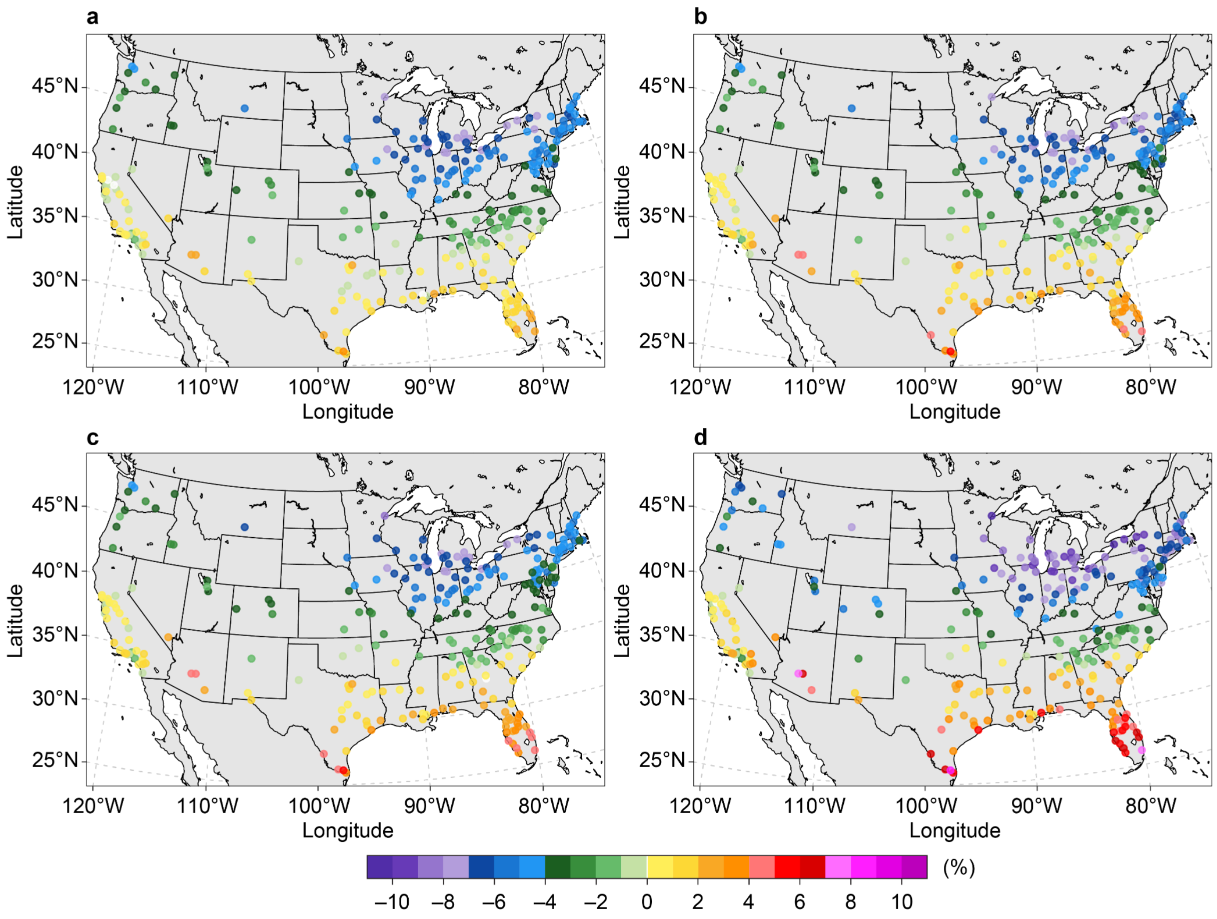

Supplementary Fig. 19. Climate change-induced annual source energy consumption change in the 2050s relative to the reference decade under four illustrative SSPX-Y scenarios and the business-as-usual power sector decarbonization scenario. a, SSP1-2.6 scenario. **b,** SSP2-4.5 scenario. **c**, SSP3-7.0 scenario. **d,** SSP5-8.5 scenario. Each point represents the relative change (%) based on the ensemble mean of the simulations driven by 10 CMIP6 models under each SSPX-Y scenario. Sources of base map: U.S. Census Bureau and Natural Earth.

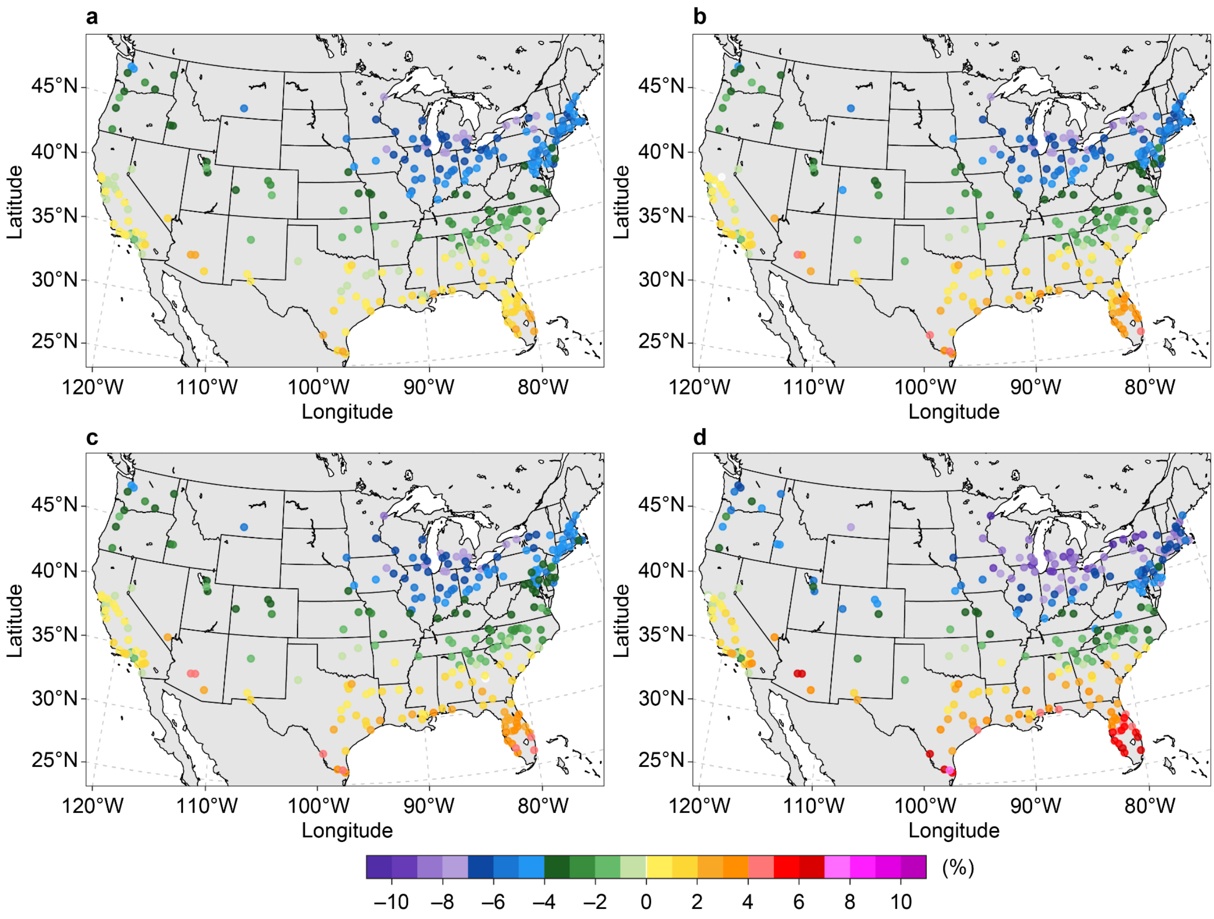

Supplementary Fig. 20. Climate change-induced annual source energy consumption change in the 2050s relative to the reference decade under four illustrative SSPX-Y scenarios and the zero-carbon power sector decarbonization scenario. a, SSP1-2.6 scenario. **b,** SSP2-4.5 scenario. **c**, SSP3-7.0 scenario. **d,** SSP5-8.5 scenario. Each point represents the relative change (%) based on the ensemble mean of the simulations driven by 10 CMIP6 models under each SSPX-Y scenario. Sources of base map: U.S. Census Bureau and Natural Earth.

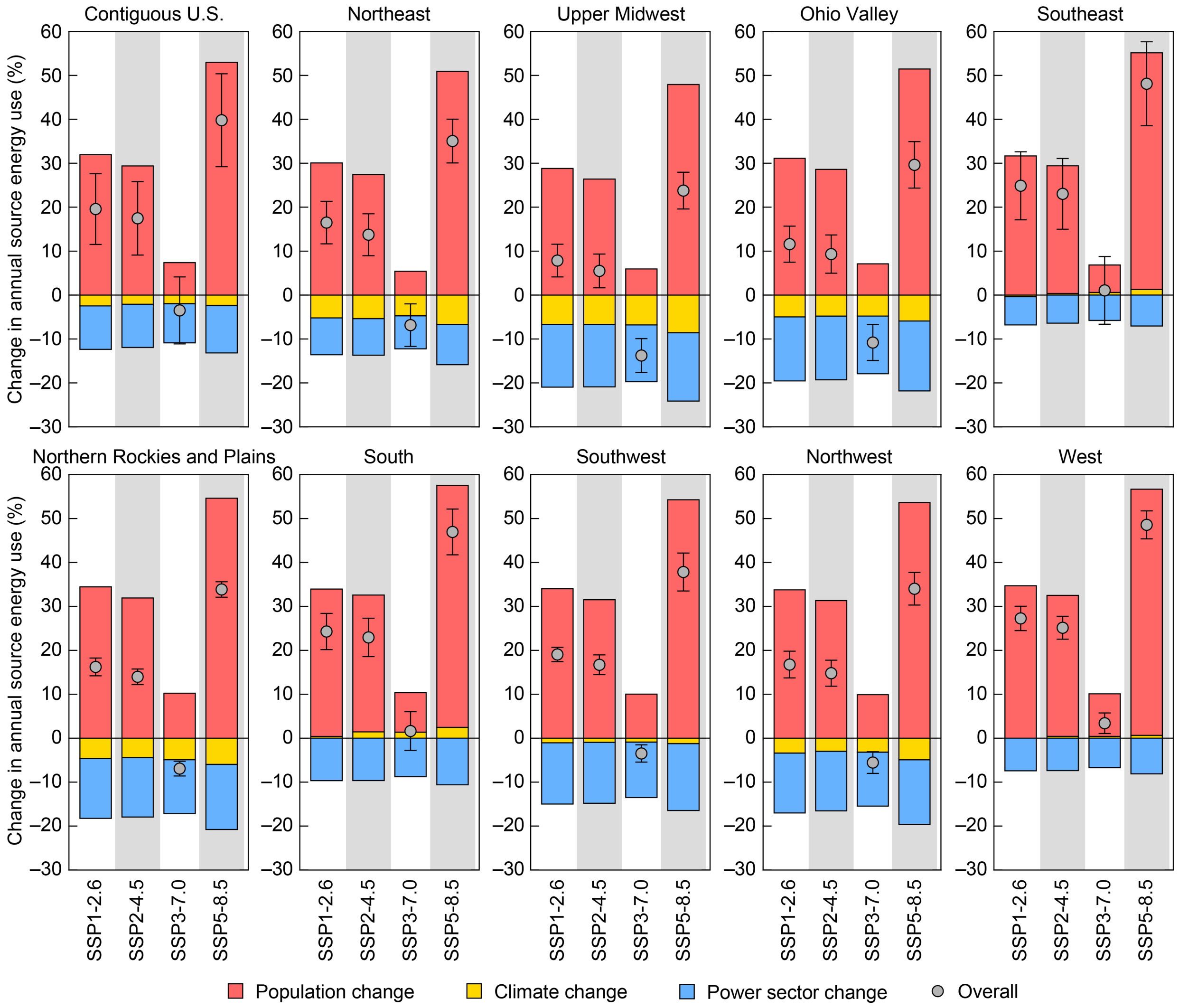

Supplementary Fig. 21. Changes in annual source energy consumption in the 2050s relative to the reference decade attributed to climate change, population change, and electric power sector decarbonization in the U.S. and nine climate regions under the business-as-usual scenario. The overall effect is the change in annual source energy consumption, which equals the sum of contributions from three drivers. Here the overall effect averaged across all urban areas within each region is presented, with error bars representing the variability (± 1 standard deviation) among urban areas.

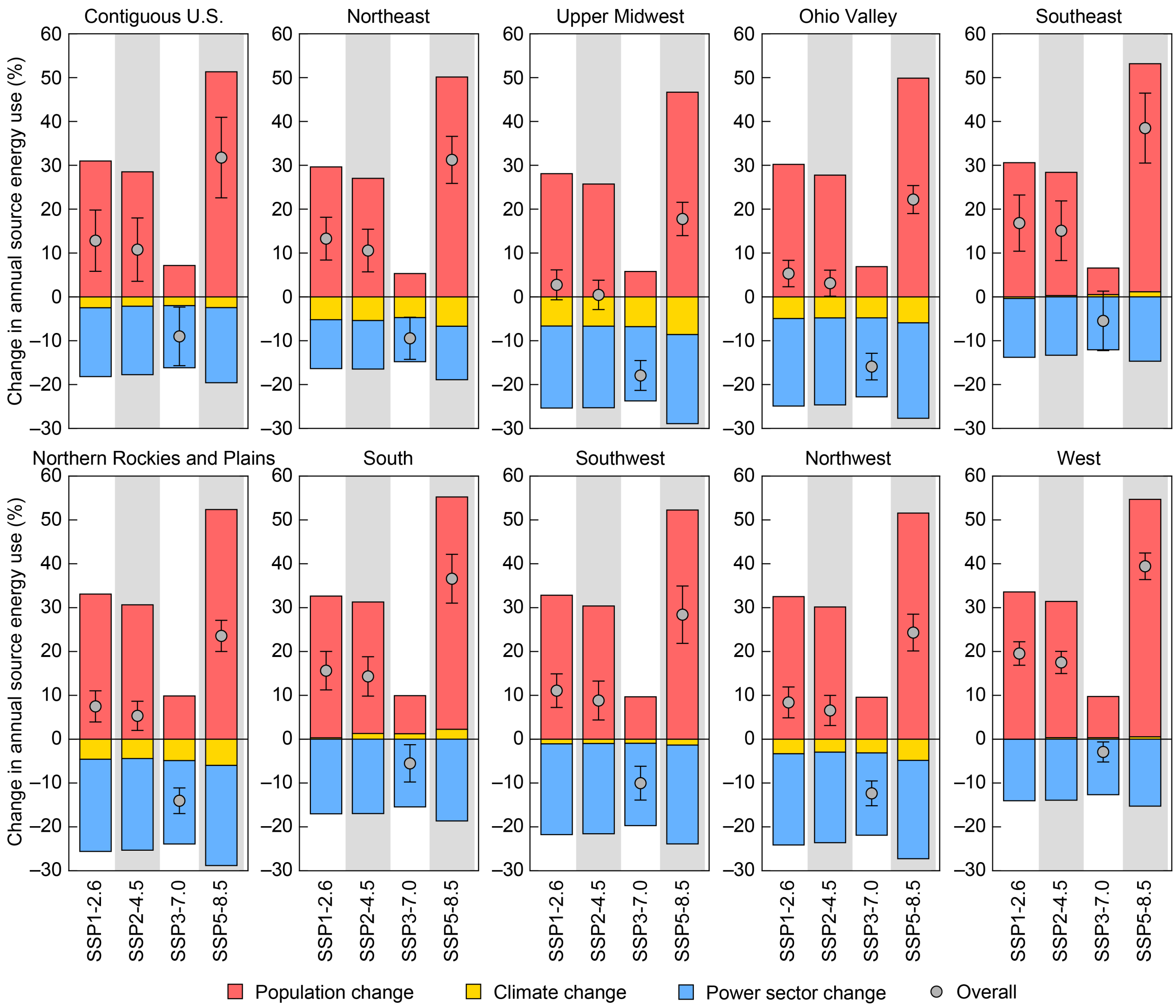

Supplementary Fig. 22. Changes in annual source energy consumption in the 2050s relative to the reference decade attributed to climate change, population change, and electric power sector decarbonization in the U.S. and nine climate regions under the zero-carbon scenario. The overall effect is the change in annual source energy consumption, which equals the sum of contributions from three drivers. Here the overall effect averaged across all urban areas within each region is presented, with error bars representing the variability (± 1 standard deviation) among urban areas.

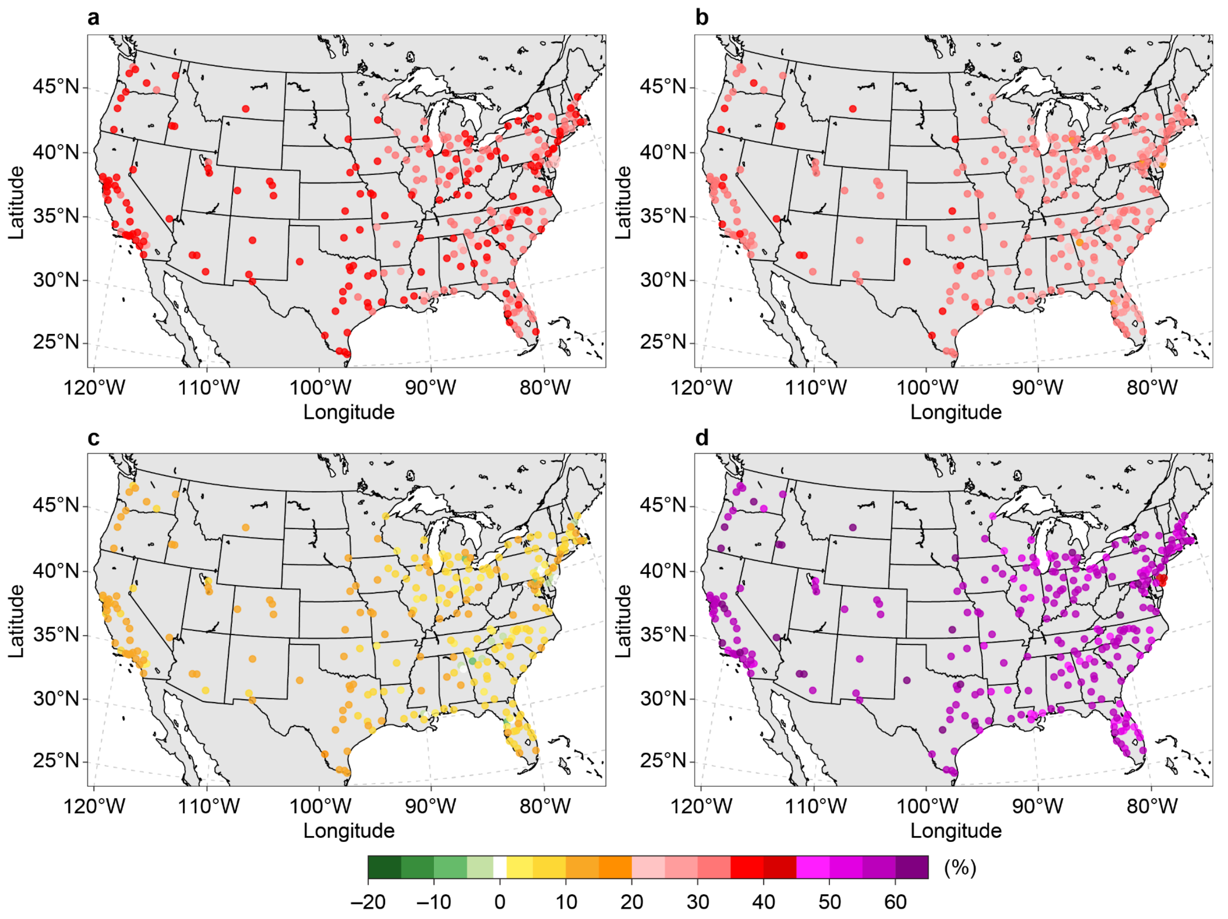

Supplementary Fig. 23. Change of city-scale population in the 2050s relative to the reference decade. a, SSP1-2.6 scenario. **b,** SSP2-4.5 scenario. **c**, SSP3-7.0 scenario. **d,** SSP5-8.5 scenario. Sources of base map: U.S. Census Bureau and Natural Earth.

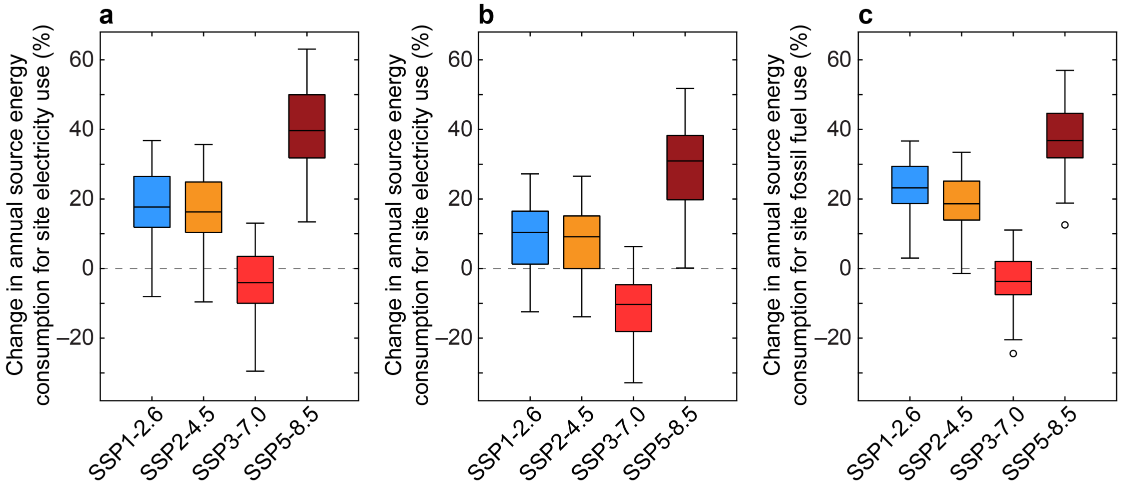

Supplementary Fig. 24. Changes in annual source energy consumption for site electricity and fossil fuel uses in the 2050s relative to the reference decade under different scenarios. **a,** Change in annual source energy consumption for site electricity use under the business-as-usual scenario. **b,** Change in annual source energy consumption for site electricity use under the zero-carbon scenario. **c**, Change in annual source energy consumption for site fossil fuel use (identical for two power section decarbonization scenarios). In each box plot, the center line shows median of changes in 277 urban areas, the box limits are upper quartile and lower quartile, the whiskers are 1.5 × interquartile range, and the points are outliners.

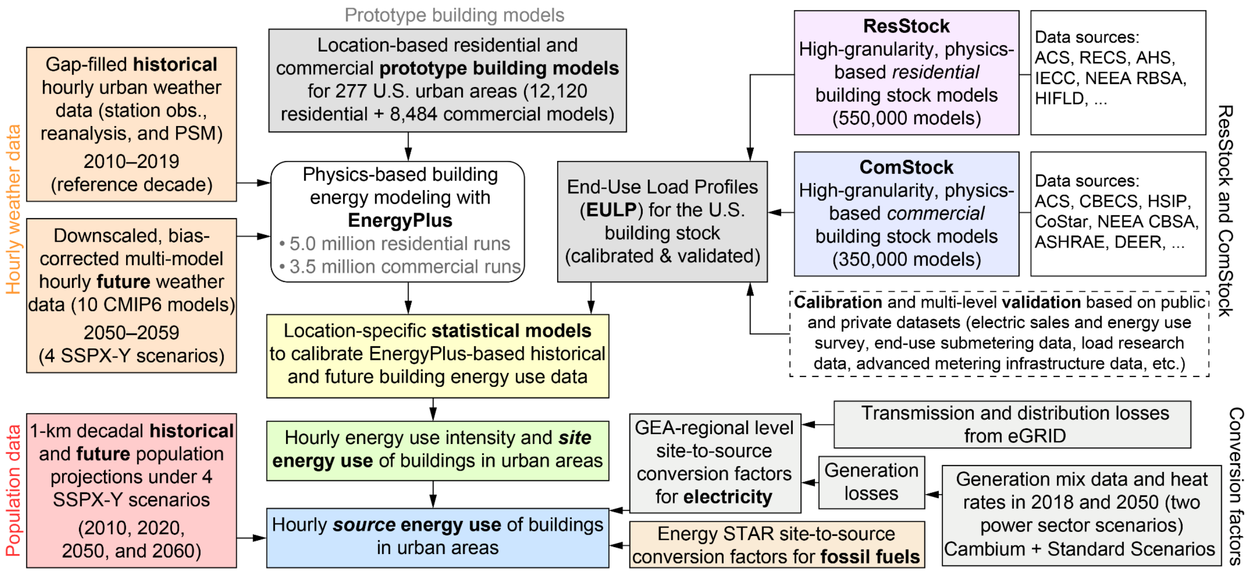

Supplementary Fig. 25. Overview of the hybrid bottom-up urban building energy modeling approach developed in this study and main data sources.

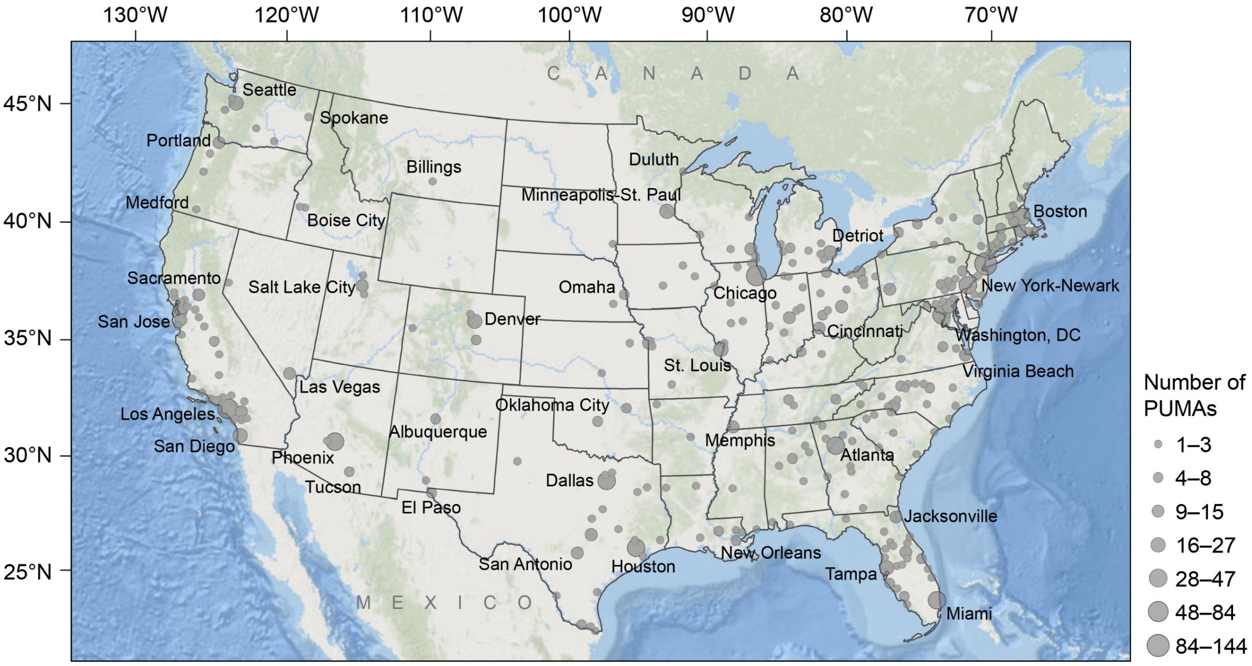

Supplementary Fig. 26. Locations of 277 urban areas in the contiguous United States. The size of each symbol represents the number of Public Use Microdata Areas (PUMAs) used to represent each urban area. Sources of ArcGIS World Ocean base map: Esri, GEBCO, DeLorme, NaturalVue, Garmin, FAO, NOAA, USGS, and EPA.

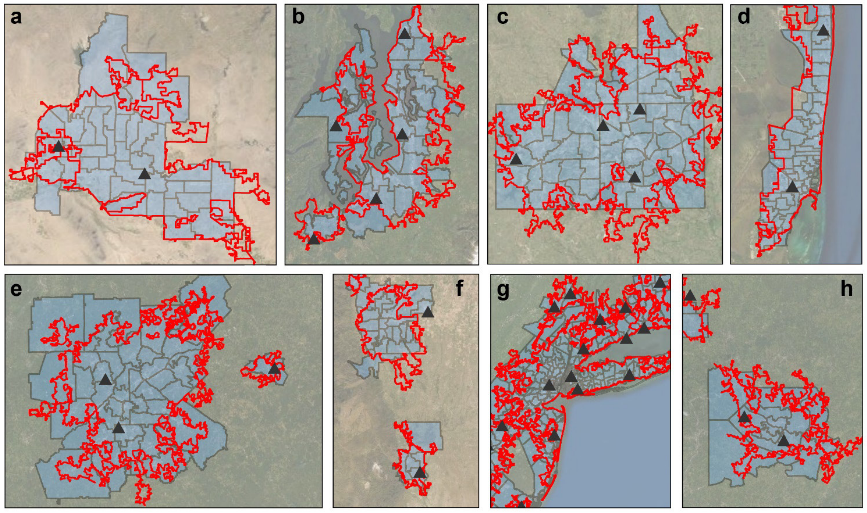

Supplementary Fig. 27. Examples of urban areas represented by PUMAs. a, Phoenix-Mesa and Avondale-Goodyear urban areas, AZ. **b**, Seattle, Bremerton, and Olympia-Lacey urban areas, WA. **c**, Dallas-Fort Worth-Arlington, Denton-Lewisville, and McKinney urban areas, TX. **d**, Miami urban area, FL. **e**, Atlanta, Gainesville, Athens-Clarke County, and Cartersville urban areas, GA. **f**. Denver-Aurora and Colorado Springs urban areas, CO. **g**, New York-Newark urban area, NY-NJ-CT and its nearby urban areas. **h**, Pittsburgh urban area, PA. Red polygons and light blue polygons are urban areas and PUMAs, respectively, based on shapefiles from U.S. Census Bureau. Black triangles in each subplot denote weather stations. Note that all subplots except for **d** show multiple urban areas. Sources of ArcGIS World Imagery base map: Esri, Maxar, Earthstar Geographics, and the GIS User Community.

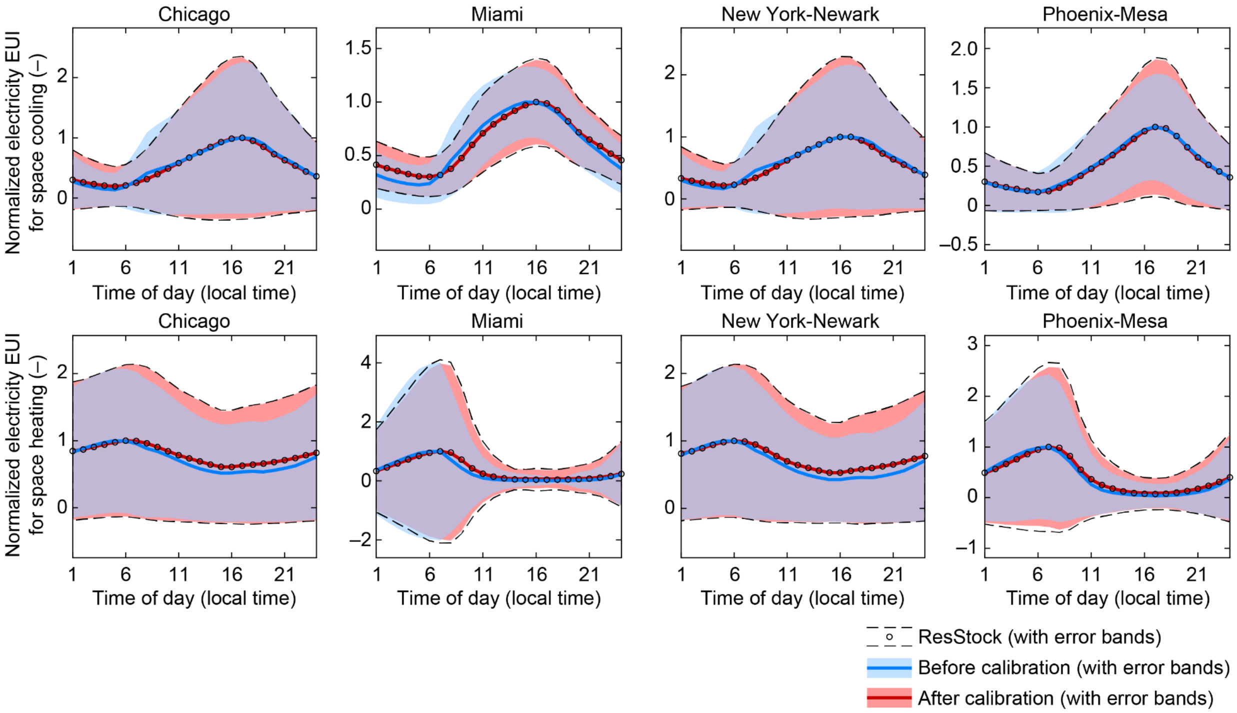

Supplementary Fig. 28. Diurnal variation of the aggregated city-scale hourly electricity energy use intensity for cooling and space heating in residential buildings (before and after calibration) and ResStock data from the EULP database in 2019 for Chicago, Miami, New York-Newark, and Phoenix-Mesa. Hourly electricity energy use intensity for each type in each urban area is normalized by its corresponding maximum value for comparison. Blue curves (with light blue error bands) and red curves (with light red error bands) show the aggregated city-scale data without and with calibration models, respectively. Data are presented as mean value ± 1 standard deviation. EUI in this figure represents energy use intensity.

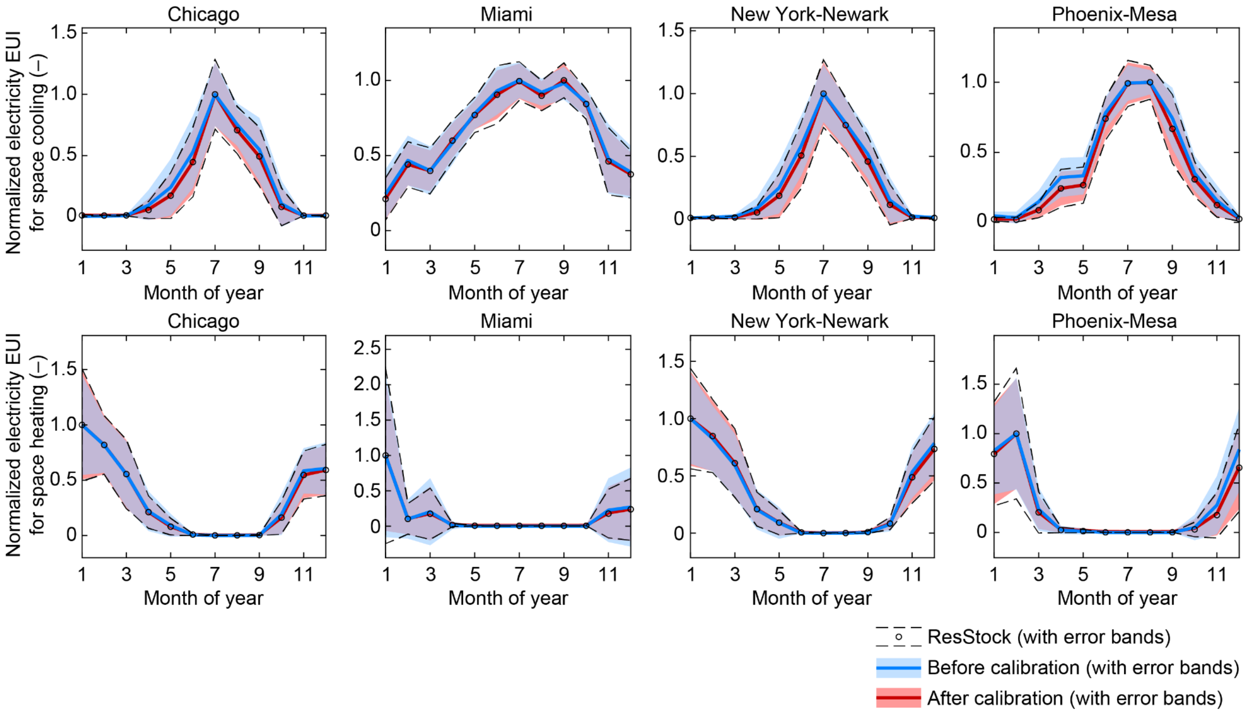

Supplementary Fig. 29. Monthly variation of the aggregated city-scale daily electricity energy use intensity for cooling and space heating in residential buildings (before and after calibration) and ResStock data from the EULP database in 2019 for Chicago, Miami, New York-Newark, and Phoenix-Mesa. Daily electricity energy use intensity for each type in each urban area is normalized by its corresponding maximum value for comparison. Blue curves (with light blue error bands) and red curves (with light red error bands) show the aggregated city-scale data without and with calibration models, respectively. Data are presented as mean value ± 1 standard deviation. EUI in this figure represents energy use intensity.

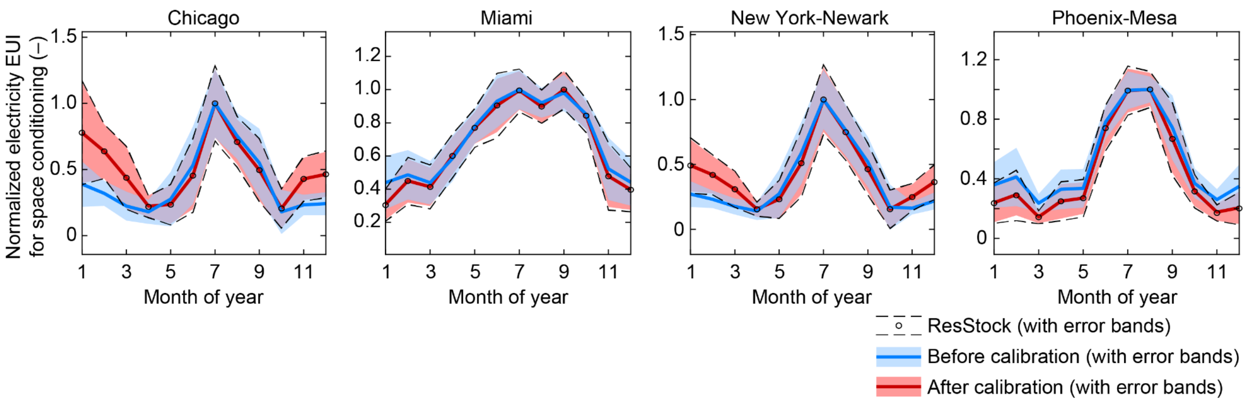

Supplementary Fig. 30. Monthly variation of the aggregated city-scale daily electricity energy use intensity for space conditioning in residential buildings (before and after calibration) and ResStock data from the EULP database in 2019 for Chicago, Miami, New York-Newark, and Phoenix-Mesa. Here space conditioning energy use is the sum of cooling energy use and space heating energy use. Daily electricity energy use intensity for each type in each urban area is normalized by its corresponding maximum value for comparison. Blue curves (with light blue error bands) and red curves (with light red error bands) show the aggregated city-scale data without and with calibration models, respectively. Data are presented as mean value ± 1 standard deviation. EUI in this figure represents energy use intensity.

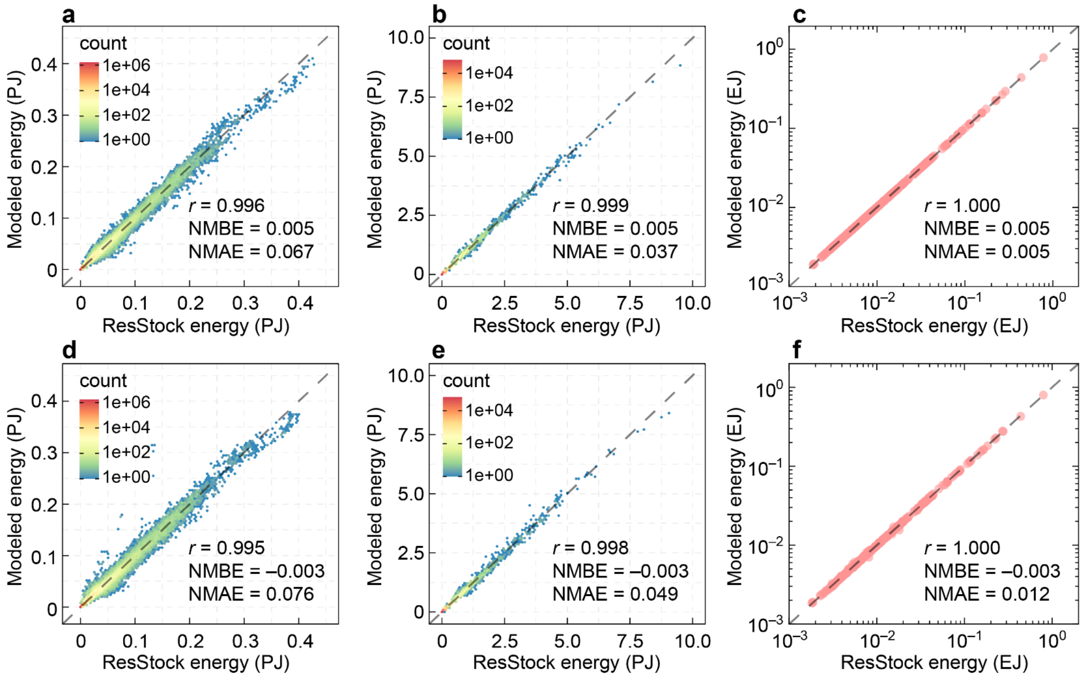

Supplementary Fig. 31. Comparison of the modeled residential energy consumption and ResStock outputs in the EULP dataset for training (2019) and testing (2018) periods. a, Hourly energy consumption in the training period (N = 2,426,520). **b,** Daily energy consumption in the training period (N = 101,105). **c**, Annual energy consumption in the training period (N = 277). **d,** Hourly energy consumption in the testing period (N = 2,426,520). **e,** Daily energy consumption in the testing period (N = 101,105). **f**, Annual energy consumption in the testing period (N = 277). Note that energy consumption data on daily and annual scales are aggregated from the hourly data. Each subplot also shows metrics for evaluation: r, NMBE, and NMAE are Pearson correlation coefficient, normalized mean bias error, and normalized mean absolute error, respectively.

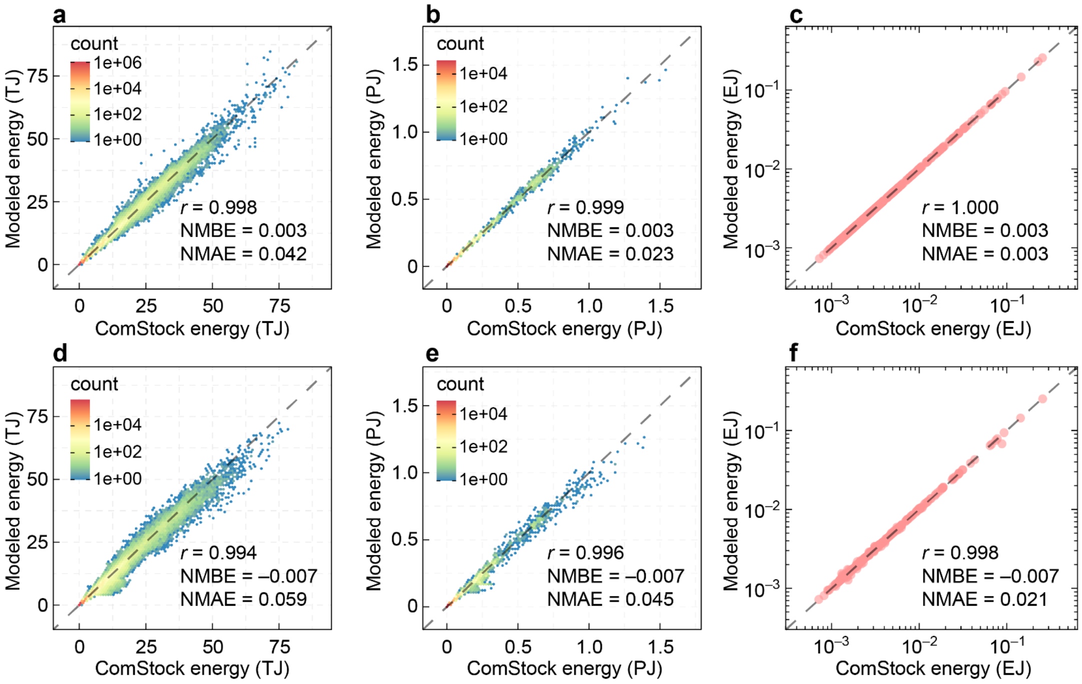

Supplementary Fig. 32. Comparison of the modeled commercial energy consumption and ComStock outputs in the EULP dataset for training (2018) and testing (TMY3) periods. a, Hourly energy consumption in the training period (N = 2,426,520). **b,** Daily energy consumption in the training period (N = 101,105). **c**, Annual energy consumption in the training period (N = 277). **d,** Hourly energy consumption in the testing period (N = 1,892,160). **e,** Daily energy consumption in the testing period (N = 78,840). **f**, Annual energy consumption in the testing period (N = 216). Note that energy consumption data on daily and annual scales are aggregated from the hourly data. Each subplot also shows metrics for evaluation: r, NMBE, and NMAE are Pearson correlation coefficient, normalized mean bias error, and normalized mean absolute error, respectively. For the testing period in TMY3, 61 urban areas are not included because the EULP TMY3 run used weather stations different from the present study (and therefore different meteorological forcing).

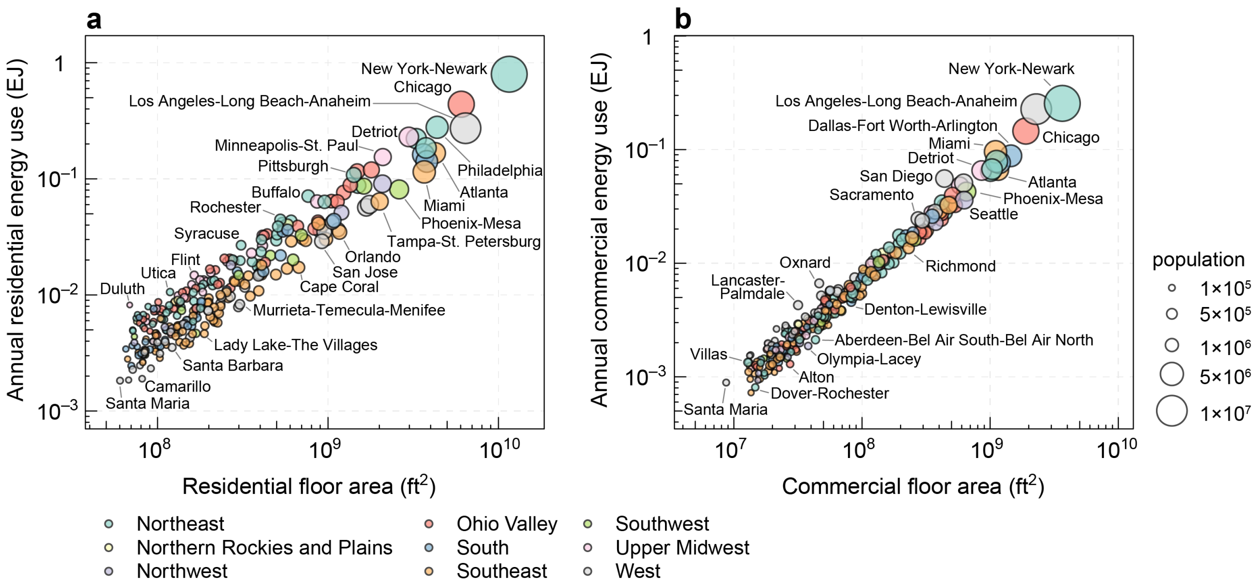

Supplementary Fig. 33. Relationships between city-scale population, floor areas, and total energy consumption of residential and commercial buildings. Data are from the EULP database for 2018. Urban areas are color-coded by their climate regions (see Supplementary Fig. 3). The size of each symbol represents the population.

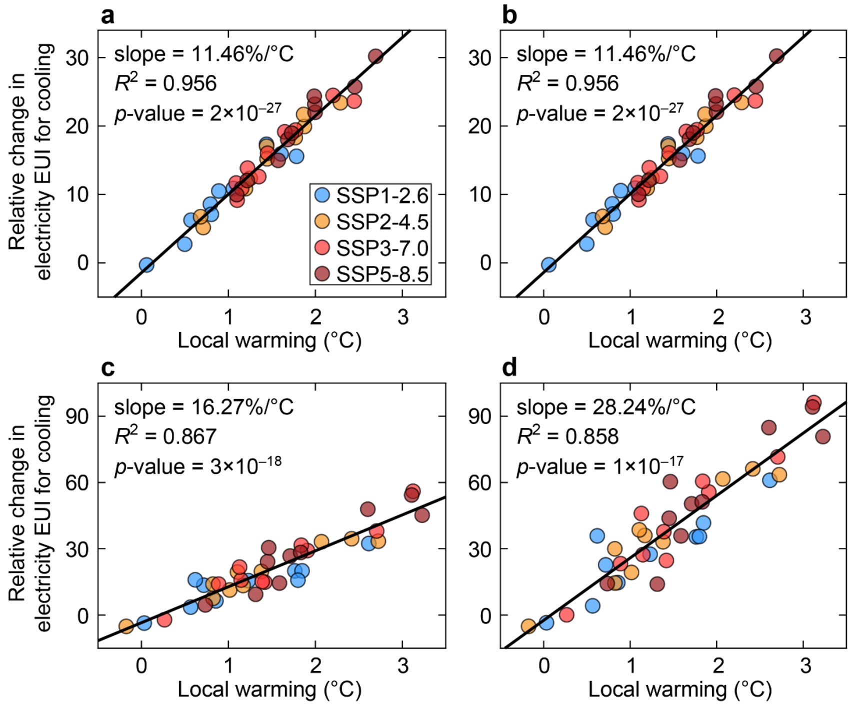

Supplementary Fig. 34. Examples of linear relationships between city-scale warming magnitude and change in annual electricity energy use intensity for cooling in the 2050s relative to the reference decade based on the projections of 10 CMIP6 models under four illustrative SSPX-Y scenarios. a, The response of electricity energy use intensity for cooling to local warming in Houston, Texas with fixed air-conditioning saturation rate as in the 2010s. **b,** The response of electricity energy use intensity for cooling to local warming in Houston, Texas with changing air-conditioning saturation rate. **c**, The response of electricity energy use intensity for cooling to local warming in Seattle, Washington with fixed air-conditioning saturation rate as in the 2010s. **d,** The response of electricity energy use intensity for cooling to local warming in Seattle, Washington with changing air-conditioning saturation rate. Black lines represent linear fits to 40 data samples (10 climate models × 4 SSPX-Y scenarios). The *p*-value is calculated based on a one-sided *F*-test. Note that the change in air-conditioning saturation rate varies in different climate models. EUI in this figure represents energy use intensity.

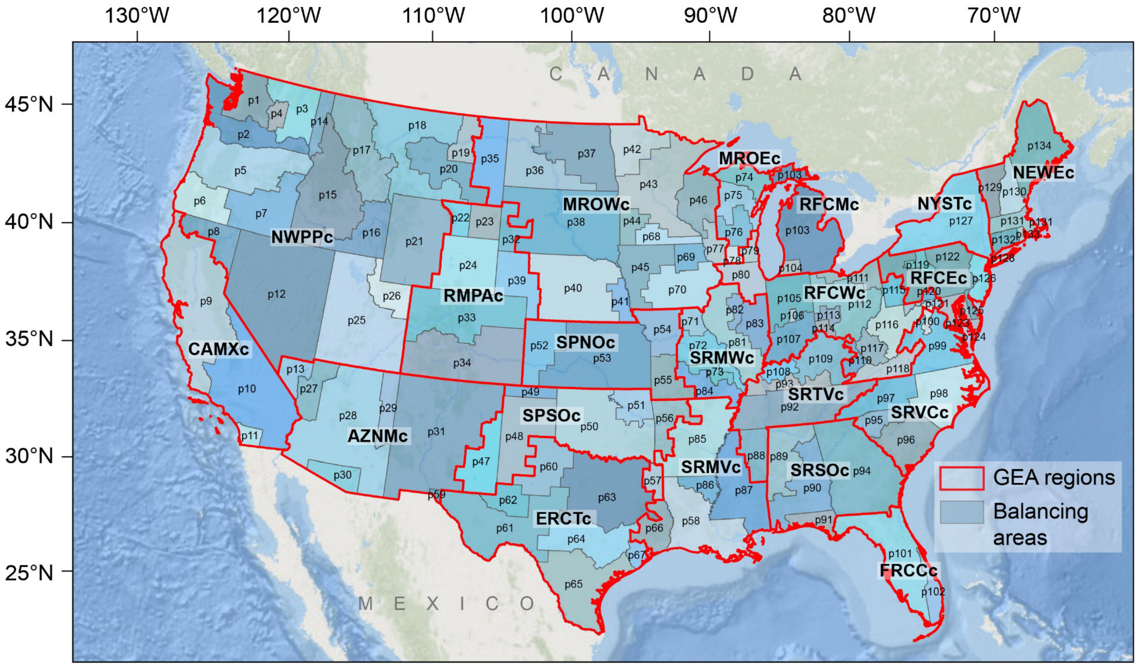

Supplementary Fig. 35. Generation and Emission Assessment (GEA) regions and balancing areas used for regional site-to-source conversion factors. GEA regions[55] are roughly consistent with the U.S. EPA's eGRID regions[72]: Electric Reliability Council of Texas (ERCTc; Texas Regional Entity), Florida Reliability Coordinating Council (FRCCc), Midwest Reliability Organization East (MROEc), Midwest Reliability Organization West (MROWc), Northeast Power Coordinating Council New England (NEWEc), Northeast Power Coordinating Council New York State (NYSTc), Reliability First Corporation East (NFCEc), Reliability First Corporation Michigan (NFCMc), Reliability First Corporation West (NFCWc), SERC Reliability Corporation Midwest (SRMWc), SERC Reliability Corporation Mississippi Valley (SRMVc), SERC Reliability Corporation South (SRSOc), SERC Reliability Corporation Tennessee Valley (SRTVc), SERC Reliability Corporation Virginia/Carolina (SRVCc), Southwest Power Pool North (SPNOc), Southwest Power Pool South (SPSOc), Western Electricity Coordinating Council California (CAMXc), Western Electricity Coordinating Council Northwest (NWPPc), Western Electricity Coordinating Council Rockies (RMPAc), and Western Electricity Coordinating Council Southwest (AZNMc). Sources of ArcGIS World Ocean base map: Esri, GEBCO, DeLorme, NaturalVue, Garmin, FAO, NOAA, USGS, and EPA.

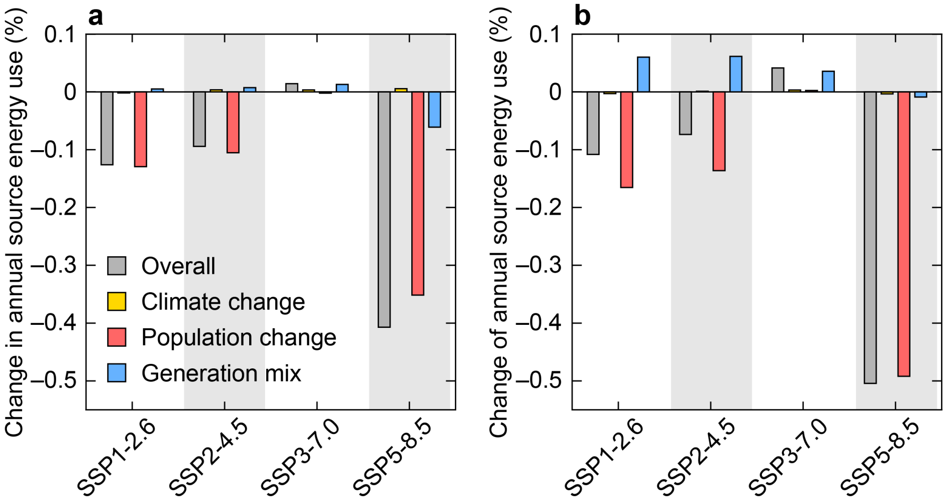

Supplementary Fig. 36. Uncertainties induced by the decomposition method used to attribute the change of source energy consumption in the 2050s relative to the reference decade. a, Business-as-usual scenario. **b,** Zero-carbon scenario. Each bar shows the difference in the contributions estimated with the elasticity-based approach and LMDI, i.e., $\Delta E^{\text{ELAS}} - \Delta E^{\text{LMDI}}$. The difference in the overall effect is equivalent to the residual term in the elasticity-based approach in Eq. (S5).

Supplementary Table 1. Shares of different technologies in the GEA regional level electricity generation (in %).

GEA	Historical (2018)				Business-as-usual future (2050)				Zero-carbon future (2050)			
	Nuclear	Coal	NG and OGS	Ren. and others	Nuclear	Coal	NG and OGS	Ren. and others	Nuclear	Coal	NG and OGS	Ren. and others
AZNMc	20.8	30.2	30.9	18.1	14.1	1.3	26.0	58.6	14.6	0	12.0	73.4
CAMXc	7.2	0	46.9	46.0	0	0	8.8	91.2	0	0	5.3	94.7
ERCTc	10.7	20.1	49.4	19.8	7.4	6.5	16.7	69.3	7.5	0	10.8	81.7
FRCCc	13.0	9.2	74.3	3.5	8.9	5.5	51.0	34.5	8.0	0	9.1	82.8
MROEc	29.6	32.7	28.7	9.1	0	11.9	16.1	72.1	0	0	11.3	88.7
MROWc	11.1	40.9	7.9	40.0	6.5	6.9	1.6	85.0	3.8	0	0.6	95.6
NEWEc	25.7	0.4	29.7	44.2	16.1	0	16.4	67.5	14.5	0	3.5	81.9
NWPPc	3.1	21.0	12.2	63.6	5.4	3.5	17.3	73.8	3.6	0	3.0	93.4
NYSTc	28.2	4.9	29.8	37.1	10.7	0	15.6	73.7	10.3	0	3.6	86.1
RFCEc	32.3	12.8	48.5	6.4	25.4	1.1	51.4	22.2	27.5	0	13.5	59.0
RFCMc	27.5	41.3	17.1	14.1	14.4	2.0	60.5	23.0	12.4	0	18.4	69.2
RFCWc	21.8	48.5	24.4	5.3	12.4	6.0	57.2	24.4	14.4	0	21.9	63.8
RMPAc	0	58.3	12.8	28.9	0	17.7	4.4	77.9	0	0	5.3	94.7
SPNOc	11.9	52.6	7.3	28.2	7.7	13.1	1.6	77.5	9.1	0	1.8	89.1
SPSOc	0	16.9	38.2	44.9	0	6.5	5.1	88.4	0	0	2.9	97.1
SRMVc	24.9	11.8	60.5	2.9	17.0	5.4	65.0	12.7	16.2	0	11.6	72.2
SRMWc	23.5	63.8	5.3	7.4	24.8	12.0	5.1	58.0	17.1	0	5.7	77.2
SRSOc	27.5	21.2	44.6	6.7	31.0	10.5	25.0	33.6	26.8	0	3.6	69.6
SRTVc	19.5	46.1	26.6	7.9	17.7	19.7	37.8	24.7	19.9	0	18.4	61.8
SRVCc	36.9	19.8	33.6	9.6	26.2	3.2	28.8	41.8	23.6	0	12.5	63.9

Note: GEA = generation and emission assessment (region), NG = natural gas, OGS = oil-gas-steam, and Ren. = renewable. The division of GEA regions is shown in Supplementary Fig. 35.

Supplementary Table 2. Global annual mean surface air temperature anomalies under different SSPX-Y scenarios in CMIP6 (mainly reproduced from ref.[14] with modifications).

	SSP1-2.6	SSP2-4.5	SSP3-7.0	SSP5-8.5
2021–2040 relative to 1995–2014	0.7 (0.4, 1.1)	0.7 (0.4, 1.2)	0.7 (0.5, 1.2)	0.8 (0.5, 1.3)
2021–2040 relative to 1850–1900	1.6 (1.1, 2.2)	1.6 (1.0, 2.3)	1.6 (1.0, 2.4)	1.7 (1.2, 2.4)
2041–2060 relative to 1995–2014	1.0 (0.6, 1.6)	1.3 (0.8, 1.9)	1.4 (0.9, 2.3)	1.7 (1.2, 2.5)
2041–2060 relative to 1850–1900	1.9 (1.2, 2.7)	2.1 (1.5, 3.0)	2.3 (1.6, 3.2)	2.6 (1.8, 3.4)
2081–2100 relative to 1995–2014	1.2 (0.6, 2.0)	2.0 (1.4, 3.0)	3.1 (2.2, 4.7)	4.0 (2.7, 5.7)
2081–2100 relative to 1850–1900	2.0 (1.3, 2.8)	2.9 (2.1, 4.0)	3.9 (2.8, 5.5)	4.8 (3.6, 6.5)
Number of models used	30	33	26	32

Note: For historical simulations, 51 models were included. The warming level is displayed as the multi-model ensemble mean (the 5th percentile, the 95th percentile).

Supplementary Table 3. Comparison of the four SSPX-Y scenarios in CMIP6 and their closest RCP scenarios in CMIP5 (reproduced from ref.[12] with modifications).

SSPX-Y scenarios	Emissions/concentrations and temperature change	Closest RCP scenarios
SSP1-2.6	Stays below 2.0°C warming relative to 1850–1900 (median) with implied net zero CO_2 emissions in the second half of the century.	RCP2.6, although RCP2.6 might be cooler for the same model settings
SSP2-4.5	Scenario approximately in line with the upper end of aggregate Nationally Determined Contributions emissions levels by 2030. CO_2 emissions remain around current levels until the middle of the century. It deviates mildly from a "no-additional-climate-policy" reference scenario and results in a best-estimate warming around 2.7°C by the end of the 21st century relative to 1850–1900.	RCP4.5 and, until 2050, also RCP6.0. Forcing under RCP6.0 was even lower than under RCP4.5 in the early decades of the 21st century.
SSP3-7.0	An intermediate-to-high reference scenario resulting from no additional climate policy under the SSP3 socio-economic development narrative. CO_2 emissions roughly double from current levels by 2100. SSP3-7.0 has particularly high non-CO_2 emissions, including high aerosols emissions.	Between RCP6.0 and RCP8.5, although SSP3-7.0 non-CO_2 emissions and aerosols are higher than in any of the RCPs.
SSP5-8.5	A high-reference scenario with no additional climate policy. CO_2 emissions roughly double from current levels by 2050.	RCP8.5, although CO_2 emissions under SSP5-8.5 are higher towards the end of the century. CH_4 emissions under SSP5-8.5 are lower than under RCP8.5. When used with the same model settings, SSP5-8.5 may result in slightly higher temperatures than RCP8.5.

Supplementary Table 4. Summary of CMIP6 general circulation models (GCMs) and Earth system models (ESMs) used in this study.

Model	Institution	Variant	Nominal resolution	Temporal resolution	Refs.
ACCESS-CM2	Commonwealth Scientific and Industrial Research Organisation and Australian Research Council Centre of Excellence for Climate System Science, Australia	r1i1p1f1	250 km	3 hr and daily	ref.[73]
BCC-CSM2-MR	Beijing Climate Center, China	r1i1p1f1	100 km	3 hr and daily	ref.[74]
CanESM5	Canadian Centre for Climate Modelling and Analysis, Canada	r1i1p2f1	500 km	3 hr, 6 hr, and daily	ref.[75]
CMCC-CM2-SR5	Centro Euro-Mediterraneo sui Cambiamenti Climatici, Italy	r1i1p1f1	100 km	3 hr	ref.[76]
CMCC-ESM2	Centro Euro-Mediterraneo sui Cambiamenti Climatici, Italy	r1i1p1f1	100 km	3 hr and daily	ref.[76]
FGOALS-g3	Chinese Academy of Sciences, China	r3i1p1f1	250 km	3 hr, 6 hr, and daily	ref.[77]
GISS-E2-1-G	Goddard Institute for Space Studies, USA	r1i1p1f2	250 km	3 hr and daily	ref.[78]
IITM-ESM	Centre for Climate Change Research, Indian Institute of Tropical Meteorology, India	r1i1p1f1	250 km	3 hr and 6 hr	ref.[79]
KACE-1-0-G	National Institute of Meteorological Sciences/Korea Meteorological Administration, Republic of Korea	r1i1p1f1	250 km	3 hr and daily	ref.[80]
MRI-ESM2-0	Meteorological Research Institute, Japan	r1i1p1f1	100 km	3 hr and daily	refs.[81,82]

Note: Temporal resolution denotes the resolution of model outputs used in this study.

Supplementary Table 5. Summary of residential and commercial building end-use categories in the outputs of the EULP used in this study.

	Major end-use category and energy source
Residential (ResStock)	Bath fan (electricity), ceiling fan (electricity), clothes dryer (electricity, natural gas, and propane), clothes washer (electricity), cooking range (electricity, natural gas, and propane), cooling (electricity), dishwasher (electricity), exterior holiday lighting (electricity), exterior lighting (electricity), extra refrigerator (electricity), fans (cooling) (electricity), fans (heating) (electricity), fireplace (natural gas), freezer (electricity), garage lighting (electricity), grill (natural gas), heating (electricity, natural gas, fuel oil, and propane), hot tub heater (electricity and natural gas), hot tub pump (electricity), house fan (electricity), interior lighting (electricity), lighting (natural gas), plug loads (electricity), pool heater (electricity and natural gas), pool pump (electricity), pumps (cooling) (electricity), pumps (heating) (electricity), range fan (electricity), recirculating pump (electricity), refrigerator (electricity), supplemental heating (electricity), vehicle (electricity), water heating (electricity, natural gas, fuel oil, and propane), and well pump (electricity)
Commercial (ComStock)	Cooling (electricity), exterior lighting (electricity), fans (electricity), heat recovery (electricity), heat rejection (electricity), heating (electricity and natural gas), interior equipment (electricity and natural gas), interior lighting (electricity), pumps (electricity), refrigerator (electricity), and water heating (electricity and natural gas)

Supplementary Table 6. Summary of the comparison between the modeled hourly residential and commercial building energy consumption and the EULP data for training and testing periods.

	Overall *r*	Overall NMBE	Overall NMAE
Residential electricity use for cooling (training)	0.992	0.007	0.114
Residential electricity use for cooling (testing)	0.990	–0.024	0.125
Residential electricity use for space heating (training)	0.991	0.009	0.126
Residential electricity use for space heating (testing)	0.986	–0.006	0.147
Commercial electricity use for cooling (training)	0.983	0.006	0.166
Commercial electricity use for cooling (testing)	0.975	0.018	0.195
Commercial electricity use for space heating (training)	0.969	0.024	0.225
Commercial electricity use for space heating (testing)	0.952	0.019	0.239
Residential electricity use for water heating (training)	0.989	0.000	0.117
Residential electricity use for water heating (testing)	0.988	–0.017	0.120
Residential natural gas use for space heating (training)	0.995	0.007	0.106
Residential natural gas use for space heating (testing)	0.993	–0.000	0.119
Commercial natural gas use for space heating (training)	0.982	0.016	0.182
Commercial natural gas use for space heating (testing)	0.971	0.000	0.202
Residential natural gas use for water heating (training)	0.993	0.000	0.093
Residential natural gas use for water heating (testing)	0.993	–0.007	0.095
Residential fuel oil use for space heating (training)	0.996	0.004	0.093
Residential fuel oil use for space heating (testing)	0.994	0.023	0.106
Residential fuel oil use for water heating (training)	0.993	0.000	0.113
Residential fuel oil use for water heating (testing)	0.993	0.010	0.116
Residential propane use for space heating (training)	0.995	0.008	0.109
Residential propane use for space heating (testing)	0.993	0.020	0.124
Residential propane use for water heating (training)	0.983	0.000	0.206
Residential propane use for water heating (testing)	0.982	0.008	0.211

Supplementary Table 7. Summary of the comparison between the modeled monthly residential and commercial building energy consumption and the EULP data for training and testing periods.

	Overall *r*	Overall NMBE	Overall NMAE
Residential electricity use for cooling (training)	1.000	0.007	0.007
Residential electricity use for cooling (testing)	1.000	–0.024	0.049
Residential electricity use for space heating (training)	1.000	0.009	0.009
Residential electricity use for space heating (testing)	0.998	–0.006	0.057
Commercial electricity use for cooling (training)	1.000	0.006	0.006
Commercial electricity use for cooling (testing)	0.997	0.018	0.071
Commercial electricity use for space heating (training)	1.000	0.024	0.024
Commercial electricity use for space heating (testing)	0.990	0.019	0.114
Residential electricity use for water heating (training)	1.000	0.000	0.000
Residential electricity use for water heating (testing)	1.000	–0.017	0.025
Residential natural gas use for space heating (training)	1.000	0.007	0.007
Residential natural gas use for space heating (testing)	0.999	–0.000	0.041
Commercial natural gas use for space heating (training)	1.000	0.016	0.016
Commercial natural gas use for space heating (testing)	0.997	0.000	0.075
Residential natural gas use for water heating (training)	1.000	0.000	0.000
Residential natural gas use for water heating (testing)	1.000	–0.007	0.018
Residential fuel oil use for space heating (training)	1.000	0.004	0.004
Residential fuel oil use for space heating (testing)	0.999	0.023	0.042
Residential fuel oil use for water heating (training)	1.000	0.000	0.000
Residential fuel oil use for water heating (testing)	1.000	0.010	0.013
Residential propane use for space heating (training)	1.000	0.008	0.008
Residential propane use for space heating (testing)	0.999	0.020	0.047
Residential propane use for water heating (training)	1.000	0.000	0.000
Residential propane use for water heating (testing)	1.000	0.008	0.023

Supplementary Table 8. Summary of the comparison between the modeled annual residential and commercial building energy consumption and the EULP data for training and testing periods.

	Overall *r*	Overall NMBE	Overall NMAE
Residential electricity use for cooling (training)	1.000	0.007	0.007
Residential electricity use for cooling (testing)	1.000	–0.024	0.029
Residential electricity use for space heating (training)	1.000	0.009	0.009
Residential electricity use for space heating (testing)	0.999	–0.006	0.031
Commercial electricity use for cooling (training)	1.000	0.006	0.006
Commercial electricity use for cooling (testing)	0.999	0.018	0.052
Commercial electricity use for space heating (training)	1.000	0.024	0.024
Commercial electricity use for space heating (testing)	0.991	0.019	0.089
Residential electricity use for water heating (training)	1.000	0.000	0.000
Residential electricity use for water heating (testing)	1.000	–0.017	0.022
Residential natural gas use for space heating (training)	1.000	0.007	0.007
Residential natural gas use for space heating (testing)	1.000	–0.000	0.018
Commercial natural gas use for space heating (training)	1.000	0.016	0.016
Commercial natural gas use for space heating (testing)	0.999	0.000	0.050
Residential natural gas use for water heating (training)	1.000	0.000	0.000
Residential natural gas use for water heating (testing)	1.000	–0.007	0.016
Residential fuel oil use for space heating (training)	1.000	0.004	0.004
Residential fuel oil use for space heating (testing)	1.000	0.023	0.027
Residential fuel oil use for water heating (training)	1.000	0.000	0.000
Residential fuel oil use for water heating (testing)	1.000	0.010	0.012
Residential propane use for space heating (training)	1.000	0.008	0.008
Residential propane use for space heating (testing)	1.000	0.020	0.028
Residential propane use for water heating (training)	1.000	0.000	0.000
Residential propane use for water heating (testing)	1.000	0.008	0.018

Supplementary Table 9. Summary of the heat rates (MMBtu/MWh) for estimating site-to-source conversion factors of electricity.

Energy technology	Heat rate (historical)	Heat rate (future)
Nuclear	10.455	10.455
Coal	10.473	10.473
Coal with carbon capture and storage	10.834	9.467
Oil-gas-steam	10.648	10.648
Natural gas combined cycle	6.363	6.363
Natural gas combined cycle with carbon capture and storage	7.159	6.170
Natural gas combustion turbine	9.717	9.717
Biomass	13.500	13.500
Bioenergy with carbon capture and storage	15.295	13.861

Note: The heat rate for coal follows the 10-year average value in the EIA's Monthly Energy Review[64]. Heat rates for other technologies are from the 2021 Annual Technological Baseline[61].

Supplementary References:

1. Wilcox, S. & Marion, W. *Users Manual for TMY3 Data Sets* (National Renewable Energy Laboratory, 2008).
2. Gelaro, R. *et al.* The Modern-Era Retrospective Analysis for Research and Applications, Version 2 (MERRA-2). *J. Clim.* **30**, 5419–5454 (2017).
3. Sengupta, M. *et al.* The National Solar Radiation Data Base (NSRDB). *Renew. Sustain. Energy Rev.* **89**, 51–60 (2018).
4. Luzio, M. D., Johnson, G. L., Daly, C., Eischeid, J. K. & Arnold, J. G. Constructing retrospective gridded daily precipitation and temperature datasets for the conterminous United States. *J. Appl. Meteorol. Climatol.* **47**, 475–497 (2008).
5. Eischeid, J. K., Pasteris, P. A., Diaz, H. F., Plantico, M. S. & Lott, N. J. Creating a serially complete, national daily time series of temperature and precipitation for the western United States. *J. Appl. Meteorol. Climatol.* **39**, 1580–1591 (2000).
6. Chen, Z., Goddard, S., Hubbard, K. G., Sorensen, W. S. & You, J. A serially complete U.S. dataset of temperature and precipitation for decision support systems. *J. Environ. Inform.* **8**, 86–99 (2015).
7. Wang, C. & Wang, Z.-H. A statistical view of the Phoenix urban heat island during the past 86 years (1933–2018). In *Central Arizona–Phoenix Long-Term Ecological Research (CAP LTER) 21st Annual All Scientists Meeting and Poster Symposium* (CAP LTER, 2019).
8. Wilson, E. J. H. *et al. End-Use Load Profiles for the U.S. Building Stock: Methodology and Results of Model Calibration, Validation, and Uncertainty Quantification* (National Renewable Energy Laboratory, 2022).
9. Henn, B., Raleigh, M. S., Fisher, A. & Lundquist, J. D. A comparison of methods for filling gaps in hourly near-surface air temperature data. *J. Hydrometeorol.* **14**, 929–945 (2013).
10. Lompar, M., Lalić, B., Dekić, L. & Petrić, M. Filling gaps in hourly air temperature data using debiased ERA5 data. *Atmosphere* **10**, 13 (2019).
11. Sunyer, M. A. *et al.* Comparison of different statistical downscaling methods to estimate changes in hourly extreme precipitation using RCM projections from ENSEMBLES. *Int. J. Climatol.* **35**, 2528–2539 (2015).
12. Chen, D. *et al. Framing, Context, and Methods*. In *Climate Change 2021: The Physical Science Basis. Contribution of Working Group I to the Sixth Assessment Report of the*

Intergovernmental Panel on Climate Change (eds Masson-Delmotte, V. et al.] (Cambridge University Press, 2021).

13. O'Neill, B. C. *et al.* The Scenario Model Intercomparison Project (ScenarioMIP) for CMIP6. *Geosci. Model Dev.* **9**, 3461–3482 (2016).
14. Lee, J.-Y. *et al.* Future Global Climate: Scenario-Based Projections and Near-Term Information. In *Climate Change 2021: The Physical Science Basis. Contribution of Working Group I to the Sixth Assessment Report of the Intergovernmental Panel on Climate Change* (eds Masson-Delmotte, V. et al.] (Cambridge University Press, 2021).
15. O'Neill, B. C. *et al.* The roads ahead: Narratives for shared socioeconomic pathways describing world futures in the 21st century. *Glob. Environ. Change* **42**, 169–180 (2017).
16. Riahi, K. *et al.* The Shared Socioeconomic Pathways and their energy, land use, and greenhouse gas emissions implications: An overview. *Glob. Environ. Change* **42**, 153–168 (2017).
17. van Vuuren, D. P. *et al.* The representative concentration pathways: an overview. *Clim. Change* **109**, 5 (2011).
18. Borrelli, P. *et al.* Land use and climate change impacts on global soil erosion by water (2015-2070). *Proc. Natl. Acad. Sci. USA* **117**, 21994–22001 (2020).
19. Pavanello, F. *et al.* Air-conditioning and the adaptation cooling deficit in emerging economies. *Nat. Commun.* **12**, 6460 (2021).
20. Taylor, Z. *National Cost-Effectiveness of the Residential Provisions of the 2018 IECC* (Pacific Northwest National Laboratory, 2021).
21. Zhang, J., Chen, Y., Xie, Y., Rosenberg, M. & Hart, R. *Energy and Energy Cost Savings Analysis of the 2018 IECC for Commercial Buildings* (Pacific Northwest National Laboratory, 2018).
22. Mendon, V. V., Lucas, R. G. & Goel, S. *Cost-Effectiveness Analysis of the 2009 and 2012 IECC Residential Provisions – Technical Support Document* (Pacific Northwest National Laboratory, 2013).
23. Taylor, Z. T., Mendon, V. V. & Fernandez, N. *Methodology for Evaluating Cost-Effectiveness of Residential Energy Code Changes* (Pacific Northwest National Laboratory, 2015).

24. Hart, R. & Liu, B. *Methodology for Evaluating Cost-Effectiveness of Commercial Energy Code Changes* (Pacific Northwest National Laboratory, 2015).
25. Zhang, J. *et al. Energy and Energy Cost Savings Analysis of the 2015 IECC for Commercial Buildings* (Pacific Northwest National Laboratory, 2015).
26. Deru, M. *et al. U.S. Department of Energy Commercial Reference Building Models of the National Building Stock* (National Renewable Energy Laboratory, 2011).
27. Thornton, B. A. *et al. Achieving the 30% Goal: Energy and Cost Savings Analysis of ASHRAE Standard 90.1-2010* (Pacific Northwest National Laboratory, 2011).
28. Goel, S. *et al. Enhancements to ASHRAE Standard 90.1 Prototype Building Models* (Pacific Northwest National Laboratory, 2014).
29. Wilson, E., Christensen, C., Horowitz, S., Robertson, J. & Maguire, J. *Energy Efficiency Potential in the U.S. Single-Family Housing Stock* (National Renewable Energy Laboratory, 2017).
30. U.S. Census Bureau. *American Community Survey 5-Year Data (2012-2016)* (U.S. Census Bureau, accessed 30 June 2022); https://www.census.gov/data/developers/data-sets/acs-5year.html.
31. U.S. Census Bureau. *2013-2017 ACS 5-year PUMS* (U.S. Census Bureau, accessed 30 June 2022); https://www.census.gov/programs-surveys/acs/microdata/documentation.html.
32. U.S. Energy Information Administration (EIA). *Residential Energy Consumption Survey (RECS): 2009 Technical Documentation-Summary* (U.S. EIA, 2013).
33. U.S. Energy Information Administration (EIA). *Residential Energy Consumption Survey (RECS): 2015 Household Characteristics Technical Documentation Summary* (U.S. EIA, 2018).
34. U.S. Census Bureau. *American Housing Survey (AHS)* (U.S. Census Bureau, accessed 30 June 2022); https://www.census.gov/AHS.
35. Northwest Energy Efficiency Alliance (NEEA). *Residential Building Stock Assessments* (NEEA, accessed 30 June 2022); https://neea.org/data/residential-building-stock-assessment.
36. Northwest Energy Efficiency Alliance (NEEA). *RBSA II Combined Database* (NEEA, accessed 30 June 2022); https://neea.org/resources/rbsa-ii-combined-database.
37. International Code Council. *2009 International Energy Conservation Code (IECC)* (International Code Council, 2009).

38. Residential Energy Service Network, Inc. *ANSI/RESNET/ICC 301-2019 Standard for the Calculation and Labeling of the Energy Performance of Dwelling and Sleeping Units using an Energy Rating Index*. (Residential Energy Service Network, Inc., 2019).
39. Wilson, E., Engebrecht-Metzger, C., Horowitz, S. & Hendron, R. *2014 Building America House Simulation Protocols* (National Renewable Energy Laboratory, 2014).
40. Bianchi, C., Zhang, L., Goldwasser, D., Parker, A. & Horsey, H. Modeling occupancy-driven building loads for large and diversified building stocks through the use of parametric schedules. *Appl. Energy* **276**, 115470 (2020).
41. Mims Frick, N. *et al. End-Use Load Profiles for the U.S. Building Stock: Market Needs, Use Cases, and Data Gaps* (National Renewable Energy Laboratory, 2019).
42. U.S. Energy Information Administration (EIA). *2012 Commercial Buildings Energy Consumption Survey (CBECS) Data* (U.S. EIA, accessed 30 June 2022); https://www.eia.gov/consumption/commercial/data/2012/.
43. CoStar. *CoStar Commercial Real Estate Public Record* (CoStar, 2022); https://www.costar.com/products/costarpublicrecord.
44. U.S. Department of Energy. *2015 U.S. Lighting Market Characterization* (U.S. Department of Energy, 2017).
45. ASHRAE. *ASHRAE: Service Life and Maintenance Cost Database* (ASHRAE, accessed 30 June 2022); http://weblegacy.ashrae.org/publicdatabase/.
46. Northwest Energy Efficiency Alliance (NEEA). *Commercial Building Stock Assessments* (NEEA, 2020); https://neea.org/data/commercial-building-stock-assessments.
47. Building Codes Assistance Project. *The Building Codes Assistance Project* (Building Codes Assistance Project, accessed 30 June 2022); http://bcapcodes.org/.
48. California Public Utilities Commission. *DEER Database - CEDARS* (California Public Utilities Commission, accessed 30 June 2022); https://cedars.sound-data.com/deer-resources/deer-database/.
49. Glasgo, B., Khan, N. & Azevedo, I. L. Simulating a residential building stock to support regional efficiency policy. *Appl. Energy* **261**, 114223 (2020).
50. Winkler, J. *et al.* Impact of installation faults in air conditioners and heat pumps in single-family homes on U.S. energy usage. *Appl. Energy* **278**, 115533 (2020).

51. White, P. R., Rhodes, J. D., Wilson, E. J. H. & Webber, M. E. Quantifying the impact of residential space heating electrification on the Texas electric grid. *Appl. Energy* **298**, 117113 (2021).
52. Gerke, B. F. *et al.* Load-driven interactions between energy efficiency and demand response on regional grid scales. *Adv. Appl. Energy* **6**, 100092 (2022).
53. Wilson, E. J. H., Harris, C. B., Robertson, J. J. & Agan, J. Evaluating energy efficiency potential in low-income households: A flexible and granular approach. *Energy Policy* **129**, 710–737 (2019).
54. Roth, J., Martin, A., Miller, C. & Jain, R. K. SynCity: Using open data to create a synthetic city of hourly building energy estimates by integrating data-driven and physics-based methods. *Appl. Energy* **280**, 115981 (2020).
55. Gagnon, P., Frazier, W., Cole, W. & Hale, E. *Cambium Documentation: Version 2021* (National Renewable Energy Laboratory, 2021).
56. Ho, J. *et al. Regional Energy Deployment System (ReEDS) Model Documentation (Version 2020)* (National Renewable Energy Laboratory, 2021).
57. Sigrin, B., Gleason, M., Preus, R., Baring-Gould, I. & Margolis, R. *The Distributed Generation Market Demand Model (dGen): Documentation* (National Renewable Energy Laboratory, 2016).
58. Energy Exemplar. *PLEXOS | Energy Market Simulation Software* (Energy Exemplar, 2022); https://www.energyexemplar.com/plexos.
59. Wang, J. *et al.* Carbon emission responsive building control: A case study with an all-electric residential community in a cold climate. *Appl. Energy* **314**, 118910 (2022).
60. Wang, J., Kontar, R. E., Jin, X. & King, J. Electrifying high-efficiency future communities: Impact on energy, emissions, and grid. *Adv. Appl. Energy* **6**, 100095 (2022).
61. Cole, W. *et al. 2021 Standard Scenarios Report: A U.S. Electricity Sector Outlook* (National Renewable Energy Laboratory, 2021).
62. U.S. Energy Information Administration (EIA). *Annual Energy Outlook 2021 with projections to 2050* (U.S. EIA, 2021).
63. Gagnon, P., Frazier, W., Hale, E. & Cole, W. *Cambium Documentation: Version 2020* (National Renewable Energy Laboratory, 2020).

64. U.S. Energy Information Administration (EIA). *May 2022 Monthly Energy Review* (U.S. EIA, 2022).
65. Troup, L. N., Fannon, D. J. & Eckelman, M. J. Spatio-temporal changes among site-to-source conversion factors for building energy modeling. *Energy Build.* **213**, 109832 (2020).
66. U.S. Environmental Protection Agency (EPA). *The Emissions & Generation Resource Integrated Database: Technical Support Document for eGRID with Year 2018 Data* (U.S. EPA, 2020).
67. U.S. Environmental Protection Agency (EPA). *The Emissions & Generation Resource Integrated Database: eGRID Technical Guide with Year 2019 Data* (U.S. EPA, 2021).
68. ENERGY STAR. *Portfolio Manager Technical Reference: Source Energy* (ENERGY STAR, 2022).
69. Lu, X. *et al.* Challenges faced by China compared with the US in developing wind power. *Nat. Energy* **1**, 1–6 (2016).
70. Peters, G. P. *et al.* Key indicators to track current progress and future ambition of the Paris Agreement. *Nat. Clim. Change* **7**, 118–122 (2017).
71. Karl, T. R. & Koss, W. J. *Regional and National Monthly, Seasonal, and Annual Temperature Weighted by Area, 1895-1983* (NOAA, 1984).
72. U.S. Environmental Protection Agency (EPA). *The Emissions & Generation Resource Integrated Database: eGRID Technical Guide with Year 2020 Data* (U.S. EPA, 2022).
73. Bi, D. *et al.* Configuration and spin-up of ACCESS-CM2, the new generation Australian Community Climate and Earth System Simulator Coupled Model. *J. South. Hemisphere Earth Syst. Sci.* **70**, 225–251 (2020).
74. Wu, T. *et al.* The Beijing Climate Center Climate System Model (BCC-CSM): the main progress from CMIP5 to CMIP6. *Geosci. Model Dev.* **12**, 1573–1600 (2019).
75. Swart, N. C. *et al.* The Canadian Earth System Model version 5 (CanESM5.0.3). *Geosci. Model Dev.* **12**, 4823–4873 (2019).
76. Cherchi, A. *et al.* Global mean climate and main patterns of variability in the CMCC-CM2 coupled model. *J. Adv. Model. Earth Syst.* **11**, 185–209 (2019).
77. Li, L. *et al.* The Flexible Global Ocean-Atmosphere-Land System Model Grid-Point Version 3 (FGOALS-g3): Description and evaluation. *J. Adv. Model. Earth Syst.* **12**, e2019MS002012 (2020).

78. Kelley, M. *et al.* GISS-E2.1: Configurations and climatology. *J. Adv. Model. Earth Syst.* **12**, e2019MS002025 (2020).
79. Swapna, P. *et al.* Long-term climate simulations using the IITM Earth System Model (IITM-ESMv2) with focus on the South Asian monsoon. *J. Adv. Model. Earth Syst.* **10**, 1127–1149 (2018).
80. Lee, J. *et al.* Evaluation of the Korea Meteorological Administration Advanced Community Earth-System model (K-ACE). *Asia-Pac. J. Atmospheric Sci.* **56**, 381–395 (2020).
81. Yukimoto, S. *et al.* The Meteorological Research Institute Earth System Model Version 2.0, MRI-ESM2.0: Description and basic evaluation of the physical component. *J. Meteorol. Soc. Jpn. Ser II* **97**, 931–965 (2019).
82. Mizuta, R. *et al.* Climate simulations using MRI-AGCM3.2 with 20-km grid. *J. Meteorol. Soc. Jpn. Ser II* **90A**, 233–258 (2012).
