# Supplementary material for: Impacts of climate change, population growth, and power sector decarbonization on urban building energy use
Source: Nat Commun. 2023 Oct 18;14:6434. doi: 10.1038/s41467-023-41458-5 (PMC10584859; doi:10.1038/s41467-023-41458-5)
Supplement: Supplementary file 3 — Description of Additional Supplementary Files [file 41467_2023_41458_MOESM3_ESM.pdf]

Description of Additional Supplementary File

File Name: Supplementary Data 1
Description: Summary of selected urban areas in the contiguous U.S.
