# Supplementary material for: Impacts of climate change, population growth, and power sector decarbonization on urban building energy use
Source: Nat Commun. 2023 Oct 18;14:6434. doi: 10.1038/s41467-023-41458-5 (PMC10584859; doi:10.1038/s41467-023-41458-5)
Supplement: Supplementary file 5 — Reporting Summary [file 41467_2023_41458_MOESM5_ESM.pdf]

nature portfolio

Corresponding author(s): Chenghao Wang

Last updated by author(s): Jul 25, 2023

Reporting Summary

Nature Portfolio wishes to improve the reproducibility of the work that we publish. This form provides structure for consistency and transparency in reporting. For further information on Nature Portfolio policies, see our Editorial Policies and the Editorial Policy Checklist.

Statistics

For all statistical analyses, confirm that the following items are present in the figure legend, table legend, main text, or Methods section.

n/a	Confirmed	
☐	☒	The exact sample size (*n*) for each experimental group/condition, given as a discrete number and unit of measurement
☒	☐	A statement on whether measurements were taken from distinct samples or whether the same sample was measured repeatedly
☐	☒	The statistical test(s) used AND whether they are one- or two-sided<br>*Only common tests should be described solely by name; describe more complex techniques in the Methods section.*
☒	☐	A description of all covariates tested
☒	☐	A description of any assumptions or corrections, such as tests of normality and adjustment for multiple comparisons
☐	☒	A full description of the statistical parameters including central tendency (e.g. means) or other basic estimates (e.g. regression coefficient) AND variation (e.g. standard deviation) or associated estimates of uncertainty (e.g. confidence intervals)
☐	☒	For null hypothesis testing, the test statistic (e.g. *F*, *t*, *r*) with confidence intervals, effect sizes, degrees of freedom and *P* value noted<br>*Give P values as exact values whenever suitable.*
☒	☐	For Bayesian analysis, information on the choice of priors and Markov chain Monte Carlo settings
☒	☐	For hierarchical and complex designs, identification of the appropriate level for tests and full reporting of outcomes
☐	☒	Estimates of effect sizes (e.g. Cohen's *d*, Pearson's *r*), indicating how they were calculated

Our web collection on statistics for biologists contains articles on many of the points above.

Software and code

Policy information about availability of computer code

Data collection	Hourly building energy use simulations are performed using EnergyPlus model version 9.0.1, which is available at https://github.com/NREL/EnergyPlus/releases/tag/v9.0.1. Parallel computing of EnergyPlus simulations was realized using MATLAB R2020b.
Data analysis	Data processing, analysis, and visualization were based on MATLAB R2020b, R 4.1.2, and R 4.2.2, and custom algorithms are available from the corresponding author upon request.

For manuscripts utilizing custom algorithms or software that are central to the research but not yet described in published literature, software must be made available to editors and reviewers. We strongly encourage code deposition in a community repository (e.g. GitHub). See the Nature Portfolio guidelines for submitting code & software for further information.

Data

Policy information about availability of data

All manuscripts must include a data availability statement. This statement should provide the following information, where applicable:

- Accession codes, unique identifiers, or web links for publicly available datasets
- A description of any restrictions on data availability
- For clinical datasets or third party data, please ensure that the statement adheres to our policy

The boundaries of urban areas and Public Use Microdata Areas (PUMAs) are extracted from the U.S. Census Bureau's TIGER/Line Shapefiles, which are available at https://www.census.gov/geographies/mapping-files/time-series/geo/tiger-line-file.html. Station-based hourly weather observations are from the Integrated Surface Database (ISD) developed by the National Centers for Environmental Information, which is available at https://www.ncei.noaa.gov/products/land-based-station/

integrated-surface-database. Historical typical meteorological year data, also derived from the ISD, are available at https://energyplus.net/weather or https://climate.onebuilding.org/sources/default.html. Historical radiation data and MERRA-2 reanalysis data are available from the National Solar Radiation Database (NSRDB) at https://nsrdb.nrel.gov/. The Coupled Model Intercomparison Project Phase 6 (CMIP6) climate projections used in this study are available through the CMIP6 Search Interface at https://esgf-node.llnl.gov/search/cmip6/. The complete dataset of the End-Use Load Profiles (EULP) for residential and commercial buildings is available at https://data.openei.org/submissions/4520. The Standard Scenarios and Cambium datasets are available at https://scenarioviewer.nrel.gov/. Downscaled high-resolution population projections are available at https://sedac.ciesin.columbia.edu/data/set/popdynamics-1-km-downscaled-pop-base-year-projection-ssp-2000-2100-rev01. Source data of all figures in the main text are provided in this paper.

Research involving human participants, their data, or biological material

Policy information about studies with human participants or human data. See also policy information about sex, gender (identity/presentation), and sexual orientation and race, ethnicity and racism.

Reporting on sex and gender	NA
Reporting on race, ethnicity, or other socially relevant groupings	NA
Population characteristics	NA
Recruitment	NA
Ethics oversight	NA

Note that full information on the approval of the study protocol must also be provided in the manuscript.

Field-specific reporting

Please select the one below that is the best fit for your research. If you are not sure, read the appropriate sections before making your selection.

☐ Life sciences ☐ Behavioural & social sciences ☒ Ecological, evolutionary & environmental sciences

For a reference copy of the document with all sections, see nature.com/documents/nr-reporting-summary-flat.pdf

Ecological, evolutionary & environmental sciences study design

All studies must disclose on these points even when the disclosure is negative.

Study description	This study uses a bottom-up modeling approach to evaluate mid-century hourly building energy consumption in 277 urban areas in the contiguous U.S. About 8.5 million EnergyPlus simulations were performed for historical and future periods. The high-granularity End-Use Load Profiles (EULP) database was used to scale up the simulated site energy use from individual building level to building stock level in each urban area. Location-specific statistical models based on historical data from the EULP dataset were used to calibrate city-scale results. Future population data and electricity generation mix data were then used to assess future changes in city-scale source energy consumption.
Research sample	This study covers 277 U.S. urban areas, which is mainly determined by the overlaps between the urbanized areas defined by the U.S. Census Bureau and the Public Use Microdata Area (PUMA). This selection allows us to cover a sufficiently large number of CONUS urban areas while considering part of the urban periphery which may undergo future urban development and potential population growth. The research sample is meant to represent the majority of U.S. urban areas.
Sampling strategy	We identified one or more PUMAs to represent each urban area. More specifically, a PUMA is selected if at least 10% of its spatial extent is within the boundaries of an urban area (representative PUMAs). In cases where a PUMA extends over multiple urban areas, we assigned it to the urban area that encompasses the largest proportion of the PUMA's spatial extent to avoid double counting. Following these procedures, we excluded urban areas without any representative PUMAs and retained 277 out of 481 CONUS urban areas in this study. The sample size is considered sufficient because these 277 urban areas encompass 92.1% of the total population in 481 CONUS urban areas.
Data collection	City-scale end-use load profiles and building stock data were collected by Chenghao Wang, Janet L. Reyna, and Henry Horsey. Cambium datasets were downloaded by Chenghao Wang. Historical weather data and future climate projection data were collected by Chenghao Wang and Ying Li. Population data were downloaded by Chenghao Wang and Zutao Ouyang.
Timing and spatial scale	All source data were collected between Nov 2020 and May 2022. Building energy simulations for the historical period are from 2010 to 2019 (to avoid the potential impact of the COVID-19 pandemic), while future simulations are from 2050 to 2059. Data from EULP, ISD, NSRDB, Standard Scenarios, and Cambium datasets cover the entire CONUS, while population projections and CMIP6 data cover the entire globe.
Data exclusions	No data were excluded from the analyses.
Reproducibility	All source data and source models used in this study are publicly available, and the EnergyPlus-based simulations of prototype building models used in this study are deterministic, which ensures the reproducibility of results.

Randomization	Not relevant. This study is based on numerical simulations of building energy use and does not involve randomization.
Blinding	Not relevant. This study is based on numerical simulations of building energy use and does not involve blinding.

Did the study involve field work? ☐ Yes ☒ No

Reporting for specific materials, systems and methods

We require information from authors about some types of materials, experimental systems and methods used in many studies. Here, indicate whether each material, system or method listed is relevant to your study. If you are not sure if a list item applies to your research, read the appropriate section before selecting a response.

Materials & experimental systems

n/a	Involved in the study	
☒	☐	Antibodies
☒	☐	Eukaryotic cell lines
☒	☐	Palaeontology and archaeology
☒	☐	Animals and other organisms
☒	☐	Clinical data
☒	☐	Dual use research of concern
☒	☐	Plants

Methods

n/a	Involved in the study	
☒	☐	ChIP-seq
☒	☐	Flow cytometry
☒	☐	MRI-based neuroimaging
